# Convergent synthesis and optical properties of near-infrared emitting bioluminescent infra-luciferins†
†Electronic supplementary information (ESI) available: General experimental details, copies of ^1^H and ^13^C NMR spectra and optical imaging details. See DOI: 10.1039/c6ra19541e



**DOI:** 10.1039/c6ra19541e

**Published:** 2017-01-16

**Authors:** James C. Anderson, Helen Grounds, Amit P. Jathoul, James A. H. Murray, Steven J. Pacman, Laurence Tisi

**Affiliations:** a Department of Chemistry , University College London , 20 Gordon Street , London , WC1H 0AJ , UK . Email: j.c.anderson@ucl.ac.uk; b School of Biosciences , University of Cardiff , Sir Martin Evans Building, Museum Avenue , Cardiff , CF10 3AX , UK; c Erba Diagnostics Mannheim , Unit 4, Cambridgeshire Business Park, Bartholomew's Walk , Ely , CB7 4EA , UK

## Abstract

Infra-luciferin, an alkene linked analogue of luciferin, gives bioluminescence emission >700 nm and has the potential to be used for multiparametric *in vivo* imaging. We report here a high yielding, scalable and convergent synthesis of infra-luciferin which will allow the synthesis of other conjugated luciferins for investigation in near-infrared bioluminescence imaging. We demonstrated this potential by using the new route to synthesise a diene linked analogue of luciferin, the fluorescent and bioluminescent properties of which were compared to those of d-luciferin and infra-luciferin. We found that extension of conjugation to a diene linker resulted in the specific bioluminescence activity being reduced by 3–4 orders of magnitude compared to d-luciferin. Analogous to its fluorescence emission spectrum, the diene linked analogue exhibited two peaks in its bioluminescence spectrum, the major one being slightly blue-shifted compared to natural d-luciferin, and a minor peak at *ca.* 800 nm. The fluorescence quantum yield and pH dependence of fluorescence were also determined.

## Abstract 2

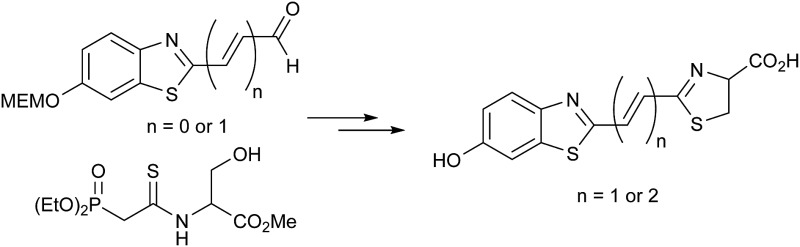
A high yielding, scalable and convergent synthesis of infra-luciferins and investigation of their potential for near-infrared bioluminescence imaging.

## Introduction

**Fig. 1:**
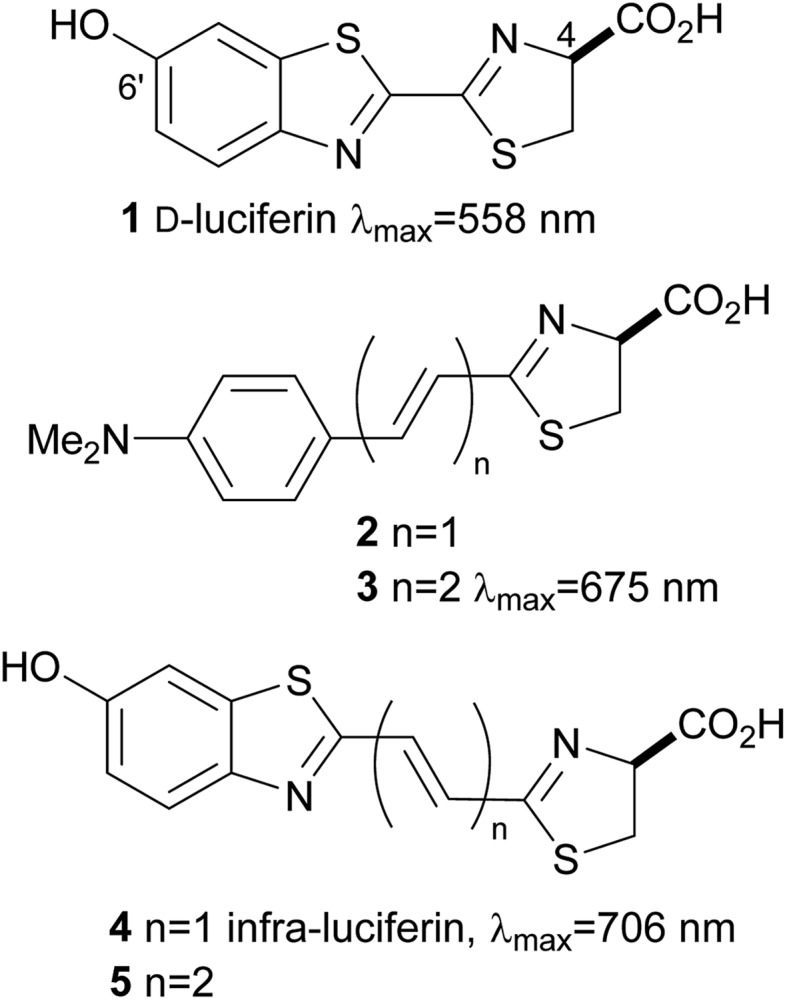
Firefly d-luciferin and synthetic extended conjugation analogues.

Noninvasive bioluminescence imaging has been critical in understanding biological phenomena in detecting disease, and is essential to probe cells and discrete molecular events within living systems. Imaging using the bioluminescence of firefly luciferase has become an important tool both *in vitro* ^1^ and *in vivo*,^2^ in particular for the tracking of tumour cells in model animals, diagnostic assays and sequencing. One of the most well studied and most widely used systems is firefly luciferase and its substrate d-luciferin (**1**) which in the presence of O_2_ and ATP emits yellow light of *λ*_max_ 558 nm (Fig. 1).^3^ This system has emerged as the most popular noninvasive imaging tool because, unlike fluorescent probes, luciferases do not require incident radiation to produce light, which means the background signal for bioluminescence imaging *in vivo* is negligible. The high signal to noise ratio lends itself to sensitive imaging applications within complex environments. In addition d-luciferin (**1**) is relatively stable, non-toxic and can penetrate most cell and tissue types following injection. The common luciferase enzymes can be purchased commercially and standard bioluminescence detectors are available in research centres.^4^

Unfortunately absorption of visible light by haemoglobin and melanin restricts image resolution and signal penetration of the light from d-luciferin (**1**).^5^ Light above *λ* 600 nm is more tissue penetrant, but despite mutations to luciferase and the synthesis of luciferin analogues it remains quite difficult to generate genuine bioluminescent emissions in the near infrared (nrIR), which would improve *in vivo* imaging.^6^ Inspired by the work of Maki and co-workers, who demonstrated the effect that extended π-conjugation can have on bioluminescence emission wavelength of synthetic luciferin derivatives such as **2** and **3**,^7^ we synthesised infra-luciferin (**4**) with an alkene linker between the benzothiazole and thiazoline fragments (Fig. 1). This gave bioluminescence emission of *λ*_max_ = 706 nm with a luciferase mutant x5 S284T and is the furthest red shifted form of bioluminescence reported to date.^8^ Akin to natural luciferin, infra-luciferin (**4**) exhibited pH dependent fluorescence spectra and exhibited bioluminescence of different colours with different engineered Fluc enzymes. Maki and coworkers noted nearly a 100 nm red shift of emitted light when adding another double bond to **2** to form **3**, together with a 3-fold drop in bioluminescence activity.^7*b*^ Similarly we wondered what effect an extra alkene linker in infra-luciferin (**4**) would have on its bioluminescence characteristics. We report here an improved synthesis of infra-luciferin (**4**) that enables the synthesis of analogues. To demonstrate this the synthesis of the diene linked luciferin **5**, akin to **3**, was accomplished and the fluorescent and bioluminescent properties of **5** were evaluated and compared to those of d-luciferin (**1**) and infra-luciferin (**4**).

## Results and discussion

**Scheme 1:**
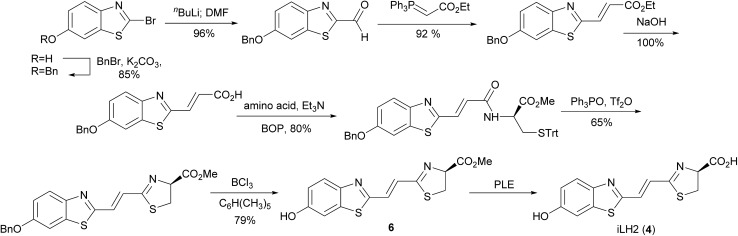
Previous reported route to infra-luciferin (**4**).

Our previously reported synthesis of infra-luciferin (**4**) gave methyl ester **6** in 17% yield over 9 steps from commercially available 6-methoxy-1,3-benzothiazol-2-amine (Scheme 1).^8^ However, this route suffered from some difficulties. The cyclisation and deprotection steps were capricious upon scale up and only small amounts of the infra-luciferin ester could be isolated. The route is linear so there are no common intermediates which could be used to synthesise other novel conjugated luciferins with additional functionality. Finally, the carboxyl group of infra-luciferin (**4**) is incredibly sensitive to epimerization, presumably due to the extended delocalization making enolisation easier. We wanted a more efficient convergent synthesis of infra-luciferin (**4**) which would also enable the rapid synthesis of other novel stretched luciferins.

**Fig. 2:**
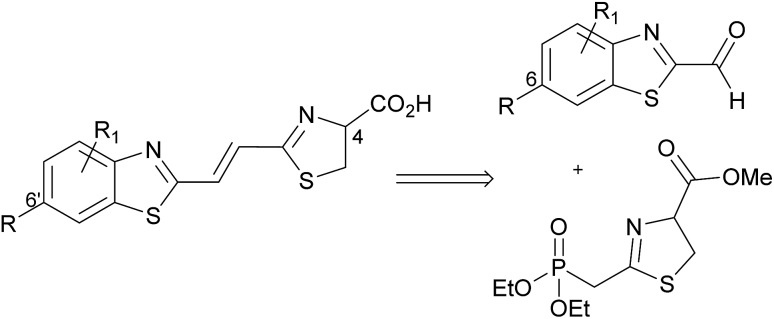
Proposed disconnection for the rapid synthesis of conjugated luciferin analogues.

We focused upon developing a racemic synthesis of infra-luciferin (**4**) as, in addition to being more facile than an enantiopure synthesis, we were interested in whether the racemic material could be used for bioluminescence imaging due to the much lower cost and availability of racemic amino acids compared to the unnatural d-amino acids. As the thiazoline in luciferin is known to be crucial for light emission^9^ we thought that this functionality could be installed using a Wittig type reaction, which would allow for a number of analogues with modified benzothiazoles or additional alkene linkers to be synthesised (Fig. 2).

**Scheme 2:**
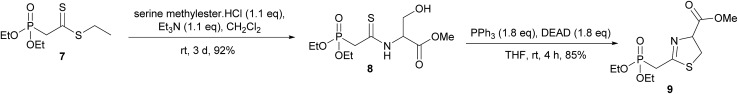
Synthesis of thiazoline **9**.

Masson *et al.* have synthesised phosphonylated thiazoline Wittig reagents, albeit without an enolisable proton, and utilised these compounds in Wittig reactions.^10^ Applying analogous methodology, ethyl 2-(diethoxyphosphoryl)ethanedithioate (**7**) (synthesised in 2 steps according to published literature procedures)^11^ was reacted with serine methyl ester to give the intermediate hydroxysubstituted thioamide (**8**) in 95% yield after column chromatography (Scheme 2). It was found that phosphonate **7** degraded upon storage even at –20 °C, however, thioamide **8** was found to be stable for at least 4 months at –20 °C, so upon isolation of **7** it was reacted immediately to give **8**. Cyclisation of **8** using a Mitsonubu reaction gave novel thiazoline **9** in 85% yield. These steps were readily scalable and could be performed on a multi-gram scale.

The synthesis of the necessary aldehyde coupling partner (Fig. 2) required the use of a protecting group for what would ultimately become the 6′-hydroxyl group of infra-luciferin (**4**). Removal of the benzyl group in our previous synthesis (Scheme 1) to form **6** had proved problematic upon scale up so different protecting groups were investigated. The methoxy-ethoxy-methyl protecting group (MEM) had been successfully used by Branchini and coworkers to protect the 6′-hydroxyl group of an *N*-acyl sulfamate analogue of luciferyl-adenylate and deprotected using trifluoroacetic acid, which did not interfere with their particular luciferin analogue.^12^ This example was with a derivative of dehydroluciferin, which is resistant to oxidative decomposition at the sensitive C-4 position because it is part of a thiazole ring. We anticipated that the acidic conditions required for removal, coupled with the expedient work up of simple evaporation, if carried out in an oxygen free atmosphere, should deliver the required thiazoline ring with the C-4 proton intact. In addition the MEM protecting group could be removed at a late stage after all other functional group manipulations had been performed, so as to minimize any oxidation or degradation of the relatively sensitive thiazoline ring.

The requisite MEM protected aldehyde **10** was prepared by standard MEM protection of 6-hydroxybenzothiazole (73%) followed by low temperature metalation of the thiazole ring and addition of DMF (87%). Attempted formation of infra-luciferin (**4**) directly by treatment with Horner–Wadsworth–Emmons reagent **9** using DBU and LiCl was unsuccessful.^9^ The use of NaH as an irreversible base in this Horner–Wadsworth–Emmons reaction gave trace quantities of protected infra-luciferin **12** and multiple unassigned products. However, the desired alkene **12** could be formed using ^*n*^BuLi in a low 23% yield.

**Scheme 3:**
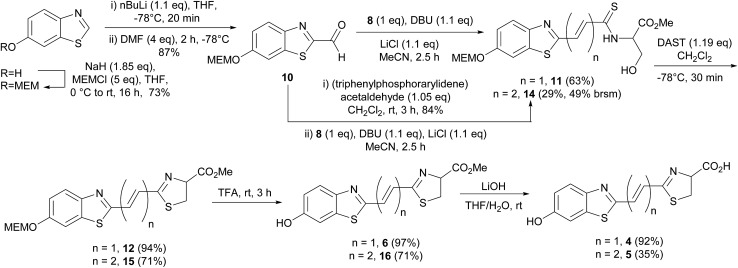
Convergent synthesis of infra-luciferin (**4**) and novel diene luciferin analogue **5**.

To overcome these problems the Horner–Wadsworth–Emmons reaction was performed using the hydroxysubstituted thioamide **8** with MEM-aldehyde **10** (Scheme 3). The use of DBU/LiCl gave the alkene **11** in 63% yield. This compound was used immediately as it was susceptible to degradation. Attempted cyclisation using DEAD/PPh_3_ only gave undesired thiazole. Under optimized conditions the use of DAST, which had previously been used by Nicolaou to form a thiazoline,^13^ gave **12** in high 94% yield and as a single pure compound after chromatography. When forming the thiazoline, and in all resultant steps, reaction solvents were sparged with argon prior to use to minimize the amount of oxygen present and hence prevent thiazole formation. Thiazole formation in d-luciferin gives dehydroluciferin which is known to inhibit the luciferase enzyme.^14^ We decided it was prudent to minimize oxidation of the thiazoline in the more conjugated derivatives. The MEM protecting group of **12** was removed using TFA to give **6** in 97% yield and finally the free acid could be isolated by treatment of **6** with LiOH to give infra-luciferin (**4**) in 92% yield. Insufficient care in reducing contact of the thiazoline with air led to contamination of either **6** or infra-luciferin (**4**) with the corresponding dehydro species which could not be separated. Small quantities (5–10%) of the dehydro materials were identified from the appearance of the thiazole proton at *δ* 8.21 (thiazole acid) and *δ* 8.41 (thiazole ester) in the ^1^H NMR spectrum. Pure **12** could be carefully treated with TFA and then LiOH under optimized reaction conditions to give uncontaminated, spectroscopically pure infra-luciferin (**4**) by ^1^H and ^13^C NMR. As a solid this material is bench stable in air for days and stable indefinitely if stored in a freezer under N_2_. Stock solutions of infra-luciferin (**4**) would gradually develop small (∼5%) amounts of dehydro material over weeks. This new route reduces the number of linear steps with an overall yield of 31%, can be scaled, and importantly the phosphonate **8** has the potential to become a common intermediate in the synthesis of other conjugated luciferins.

To further investigate extended conjugation in this analogue we prepared the diene linked compound **5** by first treating MEM-aldehyde **10** with (triphenylphosphorarylidene)acetaldehyde to give diene **13** before treatment with **8**. The Horner–Wadsworth–Emmons reaction gave a lower yield with this analogue (29%). Subsequent reactions analogous to the synthesis of infra-luciferin (**4**) gave methyl ester **16** in similar, but slightly lower yields (Scheme 3). The use of LiOH to deprotect the ester gave new analogue **5** but in an unoptimised yield of 35%. In comparison to the synthetic route to prepare infra-luciferin (**4**), as soon as extra conjugation was introduced into **13**, reactions from then on suffered from increased quantities of byproducts. These contained no alkene signals and we speculate that the substrates or reaction intermediates are susceptible to conjugate addition of nucleophilic species present in the reaction conditions. We also found the more conjugated thiazoline to be extremely sensitive to oxidation by air. Any manipulation of the final product **5** introduced varying degrees of the corresponding thiazole. Consequently saponification with LiOH was conducted swiftly and the material evaporated to dryness and used as soon as possible in the bioluminescence assay.

### Fluorescence spectra of 2′-2 alkene substituted luciferins

**Fig. 3:**
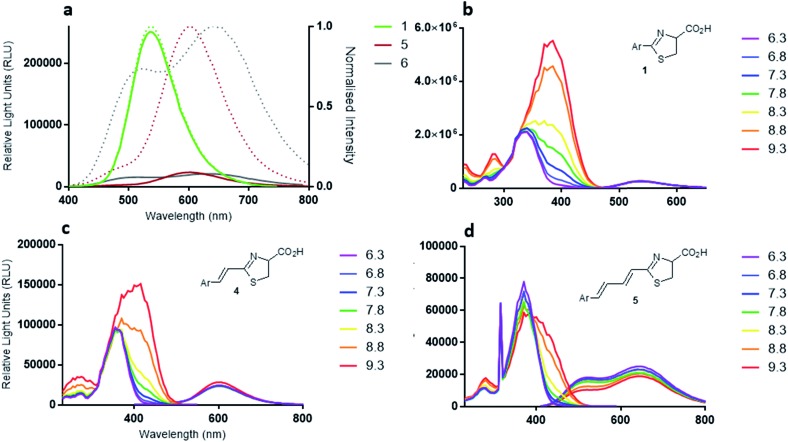
(a) Fluorescence emission spectra of d-luciferin (**1**) (green line), infra-luciferin (**4**) (red line) and **5** (black line). Dotted lines are normalized spectra, (b) pH dependence of fluorescence spectra of d-luciferin (**1**) (330 nm excitation), (c) infra-luciferin (**4**) (352 nm excitation), and (d) **5** (369 nm excitation) measured in 5 nm increments.

The fluorescence excitation and emission *λ*_max_ values of luciferin (**1**) and racemic analogues, infra-luciferin (**4**) and **5** were measured (Table 1) at pH 7.8 using a Tecan Infinite M200 instrument (Thermo Fisher Scientific, MA, USA). Natural d-luciferin (**1**) has a fluorescence excitation *λ*_max_ of 336 nm, while infra-luciferin (**4**) and **5** display *λ*_max_ 365 nm and 372 nm, respectively. Therefore, while addition of a single central alkene linker in infra-luciferin (**4**) red-shifts the excitation spectra by 29 nm, addition of another alkene (**5**) results in only a further 7 nm shift. The fluorescence emission spectra of d-luciferin (**1**), infra-luciferin (**4**) and **5** displayed *λ*_max_ of 538, 600 and 650 nm, respectively (Fig. 3a). Thus the differences between the *λ*_max_ of emission spectra are larger than the excitation spectra, with an increase of 62 nm due to the addition of one linker (**4**) and a further 50 nm with the addition of another (**5**). The stretched analogues also gave higher Stokes' shifts than d-luciferin (**1**) (202 nm). The additional single alkene in infra-luciferin (**4**) increases the Stokes' shift by 33 nm and the double alkene linker **5** increases by 76 nm relative to d-luciferin (**1**), which can be explained by higher vibrational freedom of the larger molecules. The fluorescence quantum yield of the native molecules, though not the oxyluciferin forms, can be useful,^15^ here indicating that the additional alkene linkers reduce fluorescence quantum yield of the free acids by about one half per linker.

**Table 1:** Fluorescence properties of luciferin and 2′-2 stretched racemic free acids of analogues. All compounds measured in a Tecan Infinite spectrometer with at 50 μM in TEM buffer (pH 7.8) (+/–5 nm). For quantum yields, absorptions measured at 50 μM (pH 7.8) of 100 μl, which are all less than 0.1 AU to avoid inner filter effects. Eosin Y dye used as a standard (490 nm excitation and 0.67 QY value) in basic ethanol^16^

Compound	Excitation *λ*_max_ (nm)	Emission *λ*_max_ (nm)	Fluorescence quantum yield
**1**	336	538	0.50
**4**	365	600	0.27
**5**	372	650, 505	0.17

The emission spectra of the diene linked analogue **5** displayed two peaks, (Fig. 3a) indicating the existence of two emitting species in this molecule. Interestingly, the secondary peak (505 nm) is blue-shifted compared to native d-luciferin (**1**). To examine the states of ionization of the bioluminescent molecules, pH dependence of fluorescence spectra were measured (Fig. 3b–d). Deprotonation of the 6′-hydroxyl results in red-shifts of the excitation spectra of all these molecules. Natural d-luciferin (**1**) red-shifts 40 nm when moving from pH 6.3 to 9.6 (to 385 nm). For infra-luciferin (**4**) the excitation spectra is red-shifted 47 nm, from 365 nm to 412 nm, but the red-shift for diene linked analogue **5** is 40 nm, also to *ca.* 412 nm. This suggests an upper limit to the red-shift of the excitation maxima. Using these data the p*K*_a_ of the 6′-hydroxy group of d-luciferin (**1**) was estimated to be *ca.* 8.7, and for infra-luciferin (**4**) and **5** it appeared slightly higher at 8.9. Whereas the excitation spectra become more intense with increasing pH for d-luciferin (**1**) and infra-luciferin (**4**), they reduce in intensity with diene linked analogue **5** (reversibly).

### Light yields, emission kinetics and bioluminescence spectra of wildtype *Photinus pyralis* luciferase with 2′-2 substituted luciferins

**Fig. 4:**
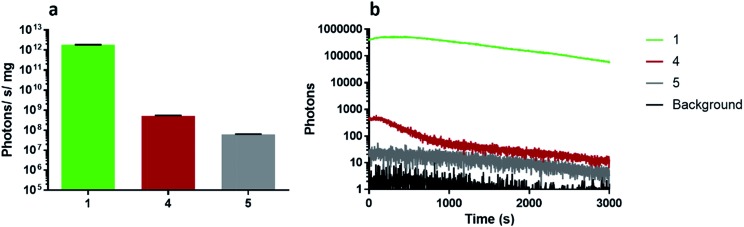
Quantification of light emission of luciferin and analogues with wild-type *Photinus pyralis* luciferase. 2 mM ATP was added to 0.17 μM pure Flucs mixed with 150 μM substrates and these were assayed in the PIO with and open filter to measure (a) specific activity, and (b) kinetics over 50 min.

Light emission with analogues infra-luciferin (**4**) and the diene linked analogue **5** was measured with purified wildtype (WT) *Photinus pyralis* luciferase (Fluc) using the PhotonIMAGER Optima 3^rd^ generation intensified CCD-containing device. This instrument has good spectral sensitivity to visible and near-infrared (nrIR) wavelengths of light. Over 50 min, WT Fluc has 3550-times less specific activity with infra-luciferin (**4**) than natural d-luciferin (**1**) and 29 000-times less with the diene linked analogue **5** (Fig. 4a). WT Fluc also displayed a prominent decay in emission from approximately 5 min until 10 min with the analogues **4** and **5** (Fig. 4b), which is not seen with d-luciferin and may be due to product inhibition. It is known that l-luciferin, the opposite enantiomer to natural d-luciferin, is a competitive luciferase inhibitor.^17^ In this work, for ease of synthesis and to obtain significant quantities of material, we developed a racemic synthesis of infra-luciferin (**4**) and **5**. Any inhibitory effect of either enantiomer of **4** or **5** was not strong enough to completely inhibit bioluminescence and it would seem that optical purity is not critical in exploring light emission of these molecules with wild-type *Photinus pyralis* luciferase. The prominent decay in emission of **4** and **5** (Fig. 4b) may be due to inhibition by one of their enantiomers,^17^ or by the corresponding oxidised luciferin or their dehydro derivatives.^18^ We know from the preparation of these compounds that extended conjugation increases the propensity towards the formation of the corresponding dehydro species *in situ* (*vide supra*).

**Fig. 5:**
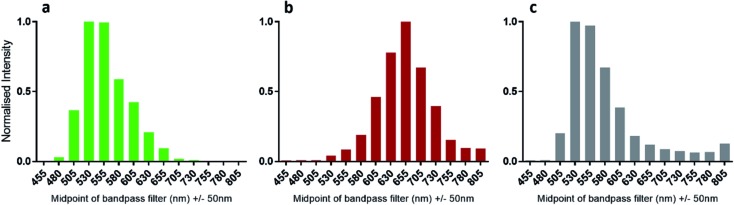
Emission spectra of luciferin and analogues with wild-type *Photinus pyralis* luciferase. (a) 0.17 μM Fluc was mixed with 150 μM d-luciferin (**1**) and 2 mM ATP and emission spectra were obtained using the PIO. (b) Infra-luciferin (**4**) in same conditions as (a). (c) 3 μM WT Fluc was used to acquire spectra with (**5**).

The bioluminescence spectra of WT Fluc with compounds **1**, **4** and **5** show that infra-luciferin (**4**) displays a 100 nm *λ*_max_ red-shift compared to **1** (Fig. 5a and b) as previously reported.^8^ However, the bioluminescence emission spectrum of diene linked analogue **5** is analogous to the fluorescence emission spectrum of the free acid of **5** in that there are two peaks, the major one being slightly blue-shifted compared to natural luciferin and then a minor peak at *ca.* 800 nm.^19^

## Conclusions

We have developed a scalable and convergent synthesis that reduces the number of linear steps to give infra-luciferin in an overall yield of 31%. The two step method to prepare **12**, by alkenation with phosphonate **8** followed by DAST mediated cyclisation, gave a higher overall yield (59%) than directly using thiazoline-phosphonate **9** (23%). The key phosphonate **8** has the potential to become a common intermediate in the synthesis of other conjugated luciferins for investigation as near-infrared bioluminescent imaging agents. This was demonstrated by the synthesis of diene linked luciferin analogue **5**. This analogue was found to have a lower quantum yield of bioluminescence than both d-luciferin (**1**) and infra-luciferin (**4**) and exhibited two peaks in its bioluminescence spectrum, the major one being slightly blue-shifted compared to natural d-luciferin, and a minor peak at *ca.* 800 nm. Although this is an extremely red shifted bioluminescent signal, it is weak and demonstrates the limitations of extended conjugation in luciferin derivatives with the WT Fluc enzyme.

Current work is aimed at developing an enantioselective synthesis of infra-luciferin (**5**) to investigate any dependence of bioluminescence on optical purity and a study of the enantiomeric purity of the material during *in vitro* and *in vivo* assays. We are also currently using this synthetic route to synthesise other infra-luciferin analogues and are developing luciferases that improve bioluminescence.

## Experimental

### Methyl 2-(2-(diethoxyphosphoryl)ethanethioamido)-3-hydroxypropanoate (**8**)

A solution of serine methyl ester (2.54 g, 16.3 mmol) in DCM (130 mL) was treated with Et_3_N (2.27 mL, 16.3 mmol) and stirred until completely dissolved (10 min). After this time a solution of ethyl 2-(diethoxyphosphoryl)ethanedithioate (**7**) (3.80 g, 14.8 mmol) in DCM (30 mL) was added dropwise. The reaction was stirred at rt for 3 d until ^31^P NMR showed no further conversion to the product. The reaction mixture was concentrated *in vacuo* to give a yellowish oil. Purification was achieved by flash column chromatography (0–10% MeOH/EtOAc) to give **8** (4.27 g, 92%) as a yellow oil. *R*_f_ = 0.42 (75% EtOAc/Pet. ether); ^1^H NMR (CDCl_3_, 300) *δ* 4.17 (m, 5H), 4.00 (dd, *J* = 11.8, 2.8, 1H), 3.82 (s, 3H), 3.49 (m, 2H), 1.35 (m, 6H); ^13^C NMR (151 MHz, CDCl_3_) *δ* 193.4, 170.0, 63.3, 61.4, 60.8, 52.9, 45.1, 16.5; ^31^P NMR (121 MHz, CDCl_3_) *δ* 21.4; IR *ν*_max_ 3301, 2983, 1744, 1435, 1231, 1049, 1023, 975 cm^–1^; HRMS (ES^+^) C_14_H_21_NOPS_2_ calcd 314.0802, found 314.0803.

### 1-[(Diethylphosphono)methyl]-(4-methoxycarbonyl-4,5-dihydro)thiazole (**9**)

A solution of **8** (1.46 g, 4.66 mmol) in THF (100 mL) was treated with PPh_3_ (2.20 g, 8.40 mmol) followed by DEAD (1.32 mL, 8.40 mmol) and stirred at rt for 4 h. Solvent was removed *in vacuo* and the resultant oil purified by flash column chromatography (5–10% MeOH/EtOAc) to give **9** (1.38 g, 85%) as an yellow oil. *R*_f_ = 0.40 (5% MeOH/EtOAc); ^1^H NMR (CDCl_3_, 300) *δ* 5.08 (dd, *J* = 15.1, 9.4, 1H), 4.25–4.03 (m, 4H), 3.80 (s, 3H), 3.71–3.50 (m, 2H), 3.31–3.10 (m, 2H), 1.33 (t, *J* = 7.1, 6H); ^13^C NMR (CDCl_3_, 150) *δ* 171.1, 165.7, 77.8, 62.9, 52.9, 36.5, 33.5, 16.5; ^31^P NMR (121 MHz, CDCl_3_) *δ* 20.6; IR *ν*_max_ 2980, 1742, 1255, 1023 cm^–1^; HRMS (ES^+^) C_10_H_19_NO_5_S calcd 296.0722, found 296.0719.

### 6-(β-Methoxyethoxymethyl ether)-2-formylbenzothiazole (**10**)

A solution of 6-(β-methoxyethoxymethyl ether)-benzothiazole (600 mg, 2.51 mmol) in THF (20 mL) was cooled to –78 °C and treated with ^*n*^BuLi (1.97 mL, 2.76 mmol, 1.40 M solution in hexane) and stirred at –78 °C for 20 min. After this time DMF (0.77 mL, 10.0 mmol) was added dropwise and the resultant solution stirred at –78 °C for 2 h. The reaction was quenched with saturated NaHCO_3(aq)_ (5 mL), taken up in EtOAc (50 mL) separated and back extracted using EtOAc (2 × 20 mL). The organic layers were dried over MgSO_4_, filtered and concentrated *in vacuo* to give **10** (585 mg, 87%) as a yellow oil. *R*_f_ = 0.32 (20% EtOAc/Pet. ether); ^1^H NMR (CDCl_3_, 600) *δ* 10.12 (s, 1H), 8.13 (d, *J* = 9.0, 1H), 7.67 (d, *J* = 2.4, 1H), 7.31 (dd, *J* = 9.0, 2.4, 1H), 5.39 (s, 2H), 3.87–3.88 (m, 2H), 3.58–3.59 (m, 2H), 3.39 (s, 3H); ^13^C NMR (CDCl_3_, 150) *δ* 185.3, 163.7, 157.9, 149.0, 138.4, 126.7, 119.2, 107.4, 94.0, 71.7, 68.2, 59.2; IR *ν*_max_ 2921, 2876, 2817, 1687, 1599, 1487, 1417, 1325, 1251, 1103, 1043, 1027, 987 cm^–1^; HRMS (ES^+^) C_12_H_14_NO_4_S calcd 268.0644, found 268.0648.

### (*E*)-Methyl 2-(3-(6-β-methoxyethoxymethyl ether-benzothiazol-2-yl)prop-2-enoylthioamido)-3-(hydroxy)propononate (**11**)

A solution of **8** (67 mg, 0.210 mmol) in MeCN (1 mL) was treated with LiCl (10 mg, 0.240 mmol), stirred 5 min, followed by DBU (36 mg, 0.240 mmol). After stirring at rt for 5 min a solution of **10** (64 mg, 0.240 mmol) in MeCN (1.5 mL) was added dropwise and stirred at rt for 2.5 h. The solution was filtered and concentrated *in vacuo*. Purification was achieved by flash column chromatography (75% EtOAc/hexanes) to give **11** (57 mg, 63%) as an orange oil. *R*_f_ = 0.31 (75% EtOAc/Pet. ether); ^1^H NMR (CDCl_3_, 600) *δ* 8.95 (br, 1H), 8.02 (d, *J* = 9.0, 1H), 7.95 (d, *J* = 15.2, 1H), 7.60 (d, *J* = 2.4, 1H), 7.45 (d, *J* = 15.1, 1H), 7.25 (dd, *J* = 9.0, 2.5, 1H), 5.38–5.40 (m, 1H), 5.36 (s, 2H), 4.20 (dd, *J* = 11.7, 3.3, 2H), 3.86–3.89 (m, 2H), 3.85 (s, 3H), 3.58–3.61 (m, 2H), 3.38 (s, 3H); ^13^C NMR (CDCl_3_, 150) *δ* 192.8, 170.0, 163.0, 156.8, 135.6, 135.0, 131.0, 123.3, 119.1, 107.4, 94.0, 77.2, 71.6, 68.2, 62.1, 60.5, 59.2, 53.2; IR *ν*_max_ 3300, 1925, 1740, 1597, 1194, 1077, 991, 822 cm^–1^; HRMS (ES^+^) C_18_H_23_N_2_O_6_S_2_ calcd 427.0998, found 427.0999.

### 6-(β-Methoxyethoxymethyl ether)-2-(2-(4-methoxycarbonyl-4,5-dihydrothiazol-2-yl)ethenyl)benzothiazole (**12**)

A solution of **11** (230 mg, 0.54 mmol) in DCM (3 mL) was cooled to –78 °C and treated with DAST (85 μL, 0.64 mmol). After stirring at –78 °C for 30 min the reaction was quenched with saturated NH_4_Cl_(aq)_ solution (3.5 mL). The layers were separated and the aqueous layer was extracted with DCM (2 × 10 mL). The combined organic layers were washed with brine (30 mL), dried (Na_2_SO_4_), filtered and concentrated. Purification was achieved by flash column chromatography (75% EtOAc/hexanes) to give **12** (206 mg, 94%) as an orange waxy solid. *R*_f_ = 0.40 (75% EtOAc/Pet. ether); ^1^H NMR (600 MHz, CDCl_3_) *δ* 7.93 (d, *J* = 9.0, 1H), 7.57 (d, *J* = 2.4, 1H), 7.41 (d, *J* = 16.2, 1H), 7.33 (d, *J* = 16.2, 1H), 7.20 (dd, *J* = 8.9, 2.4, 1H), 5.34 (s, 2H), 5.25 (t, *J* = 9.3, 1H), 3.88–3.82 (m, 5H), 3.73–3.60 (m, 2H), 3.57 (m, 2H), 3.38 (s, 3H); ^13^C NMR (CDCl_3_, 150) *δ* 171.0, 169.3, 162.6, 156.4, 149.2, 136.5, 135.0, 129.3, 124.5, 117.8, 107.4, 94.0, 78.1, 71.7, 68.0, 59.2, 53.1, 35.1; IR *ν*_max_ 2900, 1737, 1597, 1452, 1199, 1102, 991 cm^–1^; HRMS (ES^+^) C_18_H_21_N_2_O_5_S_2_ calcd 409.0892, found 409.0894.

### 6-Hydroxy-2-(2-(4*S*-methoxycarbonyl-4,5-dihydrothiazol-2-yl)ethenyl)benzothiazole (**6**)

A solution of **12** (61 mg, 0.220 mmol) in anhydrous TFA (1.5 mL) was stirred at rt for 3 h. After this time saturated NaHCO_3(aq)_ solution (15 mL) was added until gas evolution ceased, and the product extracted into EtOAc (3 × 15 mL) and separated. The organic layer was dried over NaSO_4_, stirred 30 min, filtered and concentrated *in vacuo* to give **6** (33 mg, 97%) as a pale yellow solid. Mp 153–157 °C; *R*_f_ = 0.13 (40% EtOAc/Pet. ether); ^1^H NMR (600 Hz, MeOD) *δ* 7.82 (d, *J* = 8.9, 1H), 7.41 (d, *J* = 16.1, 1H), 7.31 (d, *J* = 2.3, 1H), 7.29 (d, *J* = 16.1, 1H), 7.03 (dd, *J* = 8.9, 2.5, 1H), 5.33 (t, *J* = 9.0, 1H), 3.81 (s, 3H), 3.72 (dd, *J* = 11.3, 9.6, 1H), 3.70 (dd, *J* = 11.2, 8.6, 1H). Data agrees with that reported in the literature.^8^

### 6-Hydroxy-2-(2-(4*S*-carboxy-4,5-dihydrothiazol-2-yl)ethenyl)benzothiazole (**4**)

Argon was bubbled through the solvent for 5 min to remove oxygen before use. A suspension of **6** (50 mg, 0.156 mmol) in THF/H_2_O (2 : 1, 3.6 mL) was treated with LiOH·H_2_O (14.4 mg, 0.671 mmol) and stirred at rt for 5 min. After this time the reaction mixture was diluted with H_2_O (20 mL), extracted with EtOAc (2 × 20 mL) and Et_2_O (20 mL), the aqueous acidified using 2 M HCl to pH 3 and extracted with (2 × 20 mL) and Et_2_O (20 mL). The combined final organics were dried (Na_2_SO_4_), filtered and concentrated to give **4** (44 mg, 92%) as a bright orange solid. Mp 110 °C (dec.); ^1^H NMR (600 MHz, MeOD) *δ* 7.82 (d, *J* = 8.9, 1H), 7.40 (d, *J* = 16.1, 1H), 7.31 (d, *J* = 2.5, 1H), 7.30 (d, *J* = 16.1, 1H), 7.03 (dd, *J* = 8.9, 2.4, 1H), 5.30 (t, *J* = 9.0, 1H), 3.76–3.66 (m, 2H); ^13^C NMR (MeOD, 150) *δ* 173.3, 162.2, 158.6, 148.5, 138.1, 135.6, 128.8, 125.1, 118.1, 107.4, 78.8, 35.8; IR *ν*_max_ 3445, 3095, 1507, 1308 cm^–1^; HRMS (ES^+^) C_13_H_11_N_2_O_3_S_2_ calcd 307.0133, found 307.0212.

### (*E*)-3-(6-β-Methoxyethoxymethyl ether-benzothiazol-2-yl)-prop-2-enal (**13**)

A solution of **10** (169 mg, 0.632 mmol) in DCM (3 mL) was treated with (triphenylphosphorarylidene)acetaldehyde (202 mg, 0.663 mmol) and stirred at rt for 3 h. The crude reaction was directly loaded onto a silica column for purification. Purification was achieved by flash column chromatography (20% EtOAc/Pet. ether) to give **13** (156 mg, 84%) as a yellow solid. Mp 58–61 °C; *R*_f_ = 0.42 (40% EtOAc/Pet. ether); ^1^H NMR (CDCl_3_, 600) *δ* 9.80 (d, *J* = 7.5, 1H), 8.00 (d, *J* = 9.0, 1H), 7.71 (d, *J* = 16.0, 1H), 7.62 (d, *J* = 2.4, 1H), 7.24 (dd, *J* = 9.0, 2.4, 1H), 6.90 (dd, *J* = 16.0, 7.5, 1H), 5.37 (s, 2H), 3.87–3.88 (m, 2H), 3.58–3.59 (m, 2H), 3.39 (s, 3H); ^13^C NMR (CDCl_3_, 150) *δ* 192.6, 161.3, 156.8, 149.5, 143.7, 137.2, 133.7, 125.1, 118.3, 107.3, 94.0, 71.7, 68.1, 59.2; IR *ν*_max_ 2890, 1679, 1592, 1550, 1253, 1225, 1088, 1040, 965 cm^–1^; HRMS (CI) C_14_H_16_NO_4_S calcd 294.0800, found 294.0805.

### Methyl 2-(2*E*,4*E*-5-(6-β-methoxyethoxymethyl ether-benzothiazol-2-yl)penta-2,4-dienoylthioamido)-3-(hydroxy)propononate (**14**)

Procedure as that for **11** on a 0.142 mmol scale. Purification was achieved by flash column chromatography (75–100% EtOAc/hexanes) to give **14** (19 mg, 29%, (49% b.r.s.m.)) as an orange oil. *R*_f_ = 0.42 (70% EtOAc/Pet. ether); ^1^H NMR (CDCl_3_, 600) *δ* 8.27 (br, 1H), 7.89 (d, *J* = 8.9, 1H), 7.63 (ddd, *J* = 13.4, 6.9, 3.4, 1H), 7.54 (d, *J* = 2.4, 1H), 7.18 (dd, *J* = 8.9, 2.5, 1H), 7.12–7.16 (m, 2H), 6.60 (d, *J* = 14.5, 1H), 5.44 (dt, *J* = 7.1, 3.1, 1H), 5.34 (s, 2H), 4.22 (dd, *J* = 11.5, 3.2, 1H), 4.16 (dd, *J* = 11.4, 3.1, 1H), 3.78–3.87 (m, 2H), 3.78 (s, 3H), 3.56–3.58 (m, 2H), 3.36 (s, 3H); ^13^C NMR (CDCl_3_, 150) *δ* 194.4, 170.5, 163.9, 156.0, 149.2, 140.8, 136.2, 133.9, 133.6, 132.3, 124.0, 117.7, 107.4, 94.0, 71.7, 67.0, 62.3, 59.8, 59.2, 51.2; IR *ν*_max_ 3248, 2925, 1739, 1644, 1602, 1554, 1523, 1250, 1201, 1099, 1046, 987 cm^–1^; HRMS (ES^+^) C_20_H_25_N_2_O_6_S_2_ calcd 453.1154, found 453.1158.

### 6-β-Methoxyethoxymethyl ether-2-(4-1*E*,3*E*-(4-ethoxycarbonyl-4,5-dihydrothiazol-2-yl)buta-2,4-dienyl)benzothiazole (**15**)

Procedure as that for **12** on a 0.075 mmol scale. Purification was achieved by flash column chromatography (70% EtOAc/hexanes) to give **15** (23 mg, 71%) as an orange solid. Mp 88–90 °C; *R*_f_ = 0.25 (70% EtOAc/Pet. ether); ^1^H NMR (CDCl_3_, 600) *δ* 7.90 (d, *J* = 8.8, 1H), 7.57 (d, *J* = 2.4, 1H), 7.19 (dd, *J* = 8.8, 2.3, 1H), 7.16–7.22 (m, 1H), 7.06 (d, *J* = 15.5, 1H), 6.97 (dd, *J* = 15.4, 10.7, 1H), 6.79 (d, *J* = 15.4, 1H), 5.35 (s, 2H), 5.22 (t, *J* = 9.2, 1H), 3.86–3.88 (m, 2H), 3.83 (s, 3H), 3.66 (dd, *J* = 11.0, 9.2, 1H), 3.57–3.61 (m, 1H), 3.56–3.58 (m, 2H), 3.39 (s, 3H); ^13^C NMR (CDCl_3_, 150) *δ* 171.2, 169.6, 163.6, 156.0, 149.3, 140.3, 136.2, 134.2, 130.8, 129.1, 124.0, 117.6, 107.5, 94.0, 78.2, 71.7, 68.0, 59.2, 53.1, 34.9; IR *ν*_max_ 2920, 1736, 1650, 1597, 1251, 1200, 1163, 1099, 1046, 986 cm^–1^; HRMS (ES^+^) C_20_H_23_N_2_O_5_S_2_ calcd 435.1048, found 435.1049.

### 6-Hydroxy-2-(4-1*E*,3*E*-(4-methoxycarbonyl-4,5-dihydrothiazol-2-yl)buta-2,4-dienyl)benzothiazole (**16**)

Procedure as that for **6** on a 0.053 mmol scale. Purification was achieved by flash column chromatography (70% EtOAc/hexanes) to give **16** (16 mg, 87%) as a yellow solid. Mp 168–170 °C; *R*_f_ = 0.45 (100% EtOAc); ^1^H NMR (MeOD, 600) *δ* 7.76 (d, *J* = 8.9, 1H), 7.29 (d, *J* = 2.4, 1H), 7.25–7.31 (m, 1H), 7.08–7.18 (m, 2H), 6.99 (dd, *J* = 15.4, 2.4, 1H), 6.78 (d, *J* = 15.4, 1H), 5.27 (t, *J* = 9.0, 1H), 3.81 (s, 3H), 3.67 (dd, *J* = 11.2, 9.4, 1H), 3.63 (dd, *J* = 11.1, 8.6, 1H); ^13^C NMR (MeOD, 150) *δ* 172.6, 172.3, 164.2, 158.1, 148.4, 142.2, 135.5, 131.9, 129.3, 124.5, 117.7, 107.4, 78.6, 53.1, 35.3; IR *ν*_max_ 3029, 2951, 2921, 1733, 1610, 1596, 1556, 1271, 1201, 1172, 1046, 974 cm^–1^; HRMS (ES^+^) C_16_H_15_N_2_O_3_S_2_ calcd 347.0524, found 347.0536.

### 6-Hydroxy-2-(4-1*E*,3*E*-(4-carboxy-4,5-dihydrothiazol-2-yl)buta-2,4-dienyl) benzothiazole (**5**)

Procedure as that for **4** on a 0.043 mmol scale to give **5** (5 mg, 35%) as a brown solid (unoptimised). For characterization purposes a suspension of **16** (2 mg, 0.0057 mmol) in D_2_O (0.2 mL) was treated with LiOH (0.28 mg, 0.014 mmol) and stirred for 5 min, after this time NMR indicated complete conversion to product. ^1^H NMR (D_2_O, 600, solvent suppression of largest peak) *δ* 7.73 (d, *J* = 8.9, 1H), 7.29 (dd, *J* = 15.3, 10.6, 1H), 7.18 (dd, *J* = 15.3, 10.8, 1H), 7.15 (d, *J* = 15.4, 1H), 7.10 (d, *J* = 2.3, 1H), 6.90 (dd, *J* = 8.8, 2.3, 1H), 6.82 (d, *J* = 15.3, 1H), 5.11 (dd, *J* = 9.3, 7.9, 0.25H), 3.72 (d, *J* = 11.0, 1H), 3.52 (d, *J* = 11.1, 1H); ^13^C NMR (D_2_O, 150) *δ* 179.3, 171.9, 167.2, 161.9, 143.7, 141.9, 137.7, 134.3, 130.8, 128.1, 123.4, 121.6, 109.1, 80.1, 36.4; HRMS (ES^+^) C_15_DH_12_N_2_O_3_S_2_ calcd 334.0440, found 334.0448. There was evidence of proton/deuterium exchange at the position adjacent to the carboxylic acid resulting in the signal at *δ* 5.11 only integrating to 0.25H.
