# Supplementary material for: Convergent synthesis and optical properties of near-infrared emitting bioluminescent infra-luciferins
Source: RSC Adv. 2017 Jan 16;7(7):3975–82. doi: 10.1039/c6ra19541e (PMC5361108; doi:10.1039/c6ra19541e)

Electronic Supplementary Material (ESI) for RSC Advances.
This journal is © The Royal Society of Chemistry 2016

A Convergent Synthesis and Optical Properties of Near-infrared Emitting Bioluminescent Infra-luciferins

James C. Anderson,* Helen Grounds, Amit P. Jathoul, James A. H. Murray, Steven J. Pacman and Laurence Tisi

Supplementary Information

Index

General experimental

Biological materials and methods

Copies of ^{1}H, ^{13}C, ^{31}P NMR spectra: **8**, **9**, **10**, **11**, **12**, **6**, **4**, **13**, **14**, **15**, **16**, **5**

General Experimental Details

All manipulations were routinely carried out under an inert (Ar or N_2) atmosphere. Cooling to 0 °C was effected using an ice-water bath. Cooling to temperatures below 0 °C was effected using dry ice-acetone mixtures. For the purposes of thin layer chromatography (tlc), Merck silica-aluminium plates were used, with *uv* light (254 nm) and potassium permanganate or anisaldehyde for visualisation. For column chromatography Merck Geduran® Si 60 silica gel was used. Butyl lithium solutions were standardised with diphenyl acetic acid.

All reagents were used as received unless stated. Anhydrous solvents (THF, DCM, MeCN) were obtained from a solvent tower, where degassed solvent was passed through two columns of activated alumina and a 7 micron filter under 4 bar pressure. 6-(*β*-Methoxyethoxymethylether)benzothiazole and ethyl *O*,*O*-diethylphosphonodithioacetate (**8**) were synthesised according to the published procedures.[1, 2]

Melting points are uncorrected and were recorded on a Griffin melting point machine. All NMR data was collected using a Bruker AMX 300 MHz or a Bruker AVANCE III 600 MHz as stated. All chemical shifts (δ) are reported in parts per million (ppm) relative to residual solvent peaks. Reference values for residual solvents were taken as δ = 7.27 ($CDCl_3$), 2.51 (DMSO –*d*6), 3.30 (MeOD- *d*4) ppm for ^1H NMR and δ = 77.2 ($CDCl_3$), 39.5 (DMSO –*d*6), 49.0 (MeOD- *d*4) ppm for ^{13}C NMR. Coupling constants (*J*) are reported in Hertz and are recorded as observed in the spectrum without averaging. Infrared spectra were recorded using a Bruker Alpha ATR spectrometer. Mass spectrometry data was collected on a Micromass LCT Premier XE (ESI) or Thermo Finnigan Mat900xp (EI/CI) instrument.

Biological materials and methods

Optical properties

Fluorescence quantum yields of analogues (Table 1). Fluorescence spectra of 50uM analogues were measured as above and furthermore, the emission of 1uM Eosin Yellow dye (BDH Lab Supplies, Poole, UK) in basic ethanol was measured at an excitation wavelength of 490nm. Furthermore, absorption readings of 50uM analogues were also measured and used to derive fluorescence quantum yields[3] of analogues at pH 7.8 in TEM buffer.

pH dependence of fluorescence spectra of D-luciferin (1) and analogues 4 and 5 (Fig 3). 10mg/ml analogues in 50% DMSO were dissolved in 500ul TEM buffer (pH 7.8) for concentrations of 500uM

each. They were further diluted to 50uM in different solutions of TEM of differing pH values (pH 6.3, 6.8, 7.3, 7.8, 8.3, 8.8 & 9.3). The solutions were checked for pH after being made up with analogues (values displayed in data). Data were acquired in 2 instruments: the Varian Cary Eclipse Spectrofluorometer and also the Tecan Infinite M200 Spectrofluorometer in triplicate of triplicates on each machine.

Bioluminescence specific activities of luciferin analogues with wild-type firefly luciferase (Fig 4). Bioluminescence assays were conducted using the PhotonIMAGER Optima (Biospace Labs, Paris, France) at 26-28°C. 50ul of 6mM ATP was dispensed onto 50ul substrates mixed with different luciferases in triplicate. Experiments were carried out several times with 0.05uM enzymes for native luciferin and 0.5uM enzymes for analogues.

Bioluminescence spectra of wild-type luciferase enzymes with luciferin and analogues. 1-10mM enzymes were used to determine bioluminescence spectra with luciferin and analogues at differing pH. The spectrum of Eosin Yellow in basic ethanol was compared to the absolute spectrum[4] to correct spectra for PMT sensitivity.

References

1) E. Pfund, T. Lequeux, S. Masson and M. Vazeux, *Org. Lett.,* 2002, **4**, 843.

2) C. S. Marvel, P. de Radzitzky and J. J. Brader, *J. Am. Chem. Soc.,* 1955, **77**, 5997.

3) *Principles of Fluorescence Spectroscopy*, Editor: Joseph R. Lakowicz, Springer US, 2006. ISBN: 978-0-387-31278-1.

4) http://omlc.org/spectra/PhotochemCAD/html/061.html (accessed June 2016)

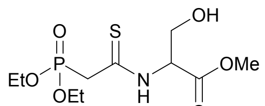

8

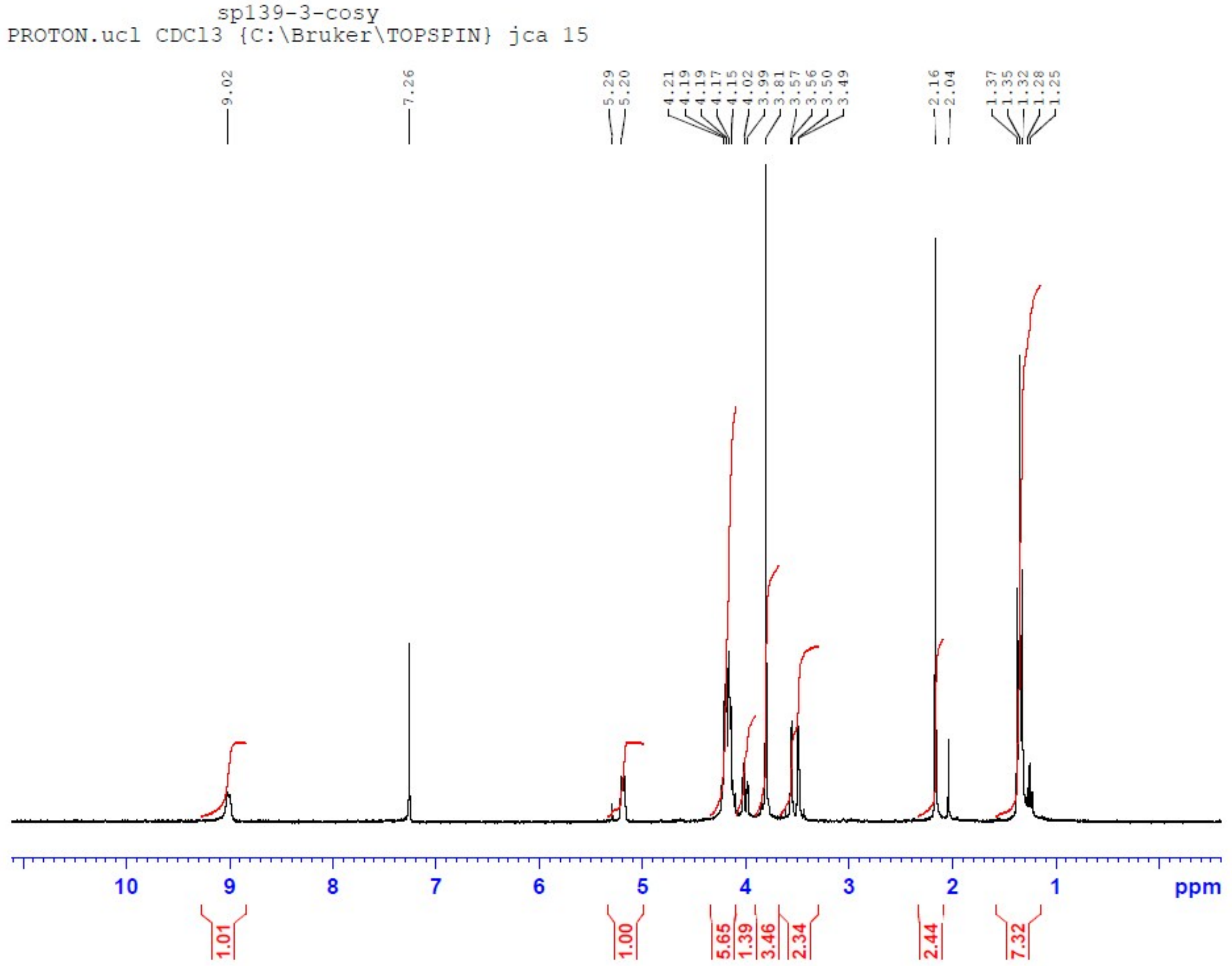


```
Current Data Parameters
NAME          sp139-3-cosy
EXPNO                   10
PROCNO                   1

F2 - Acquisition Parameters
Date_             20150724
Time                 11.05
INSTRUM              spect
PROBHD   5 mm QNP 1H/13
PULPROG               zg30
TD                   49382
SOLVENT              CDCl3
NS                       4
DS                       0
SWH           6172.839 Hz
FIDRES        0.125002 Hz
AQ           3.9999421 sec
RG                  2580.3
DW            81.000 usec
DE              6.50 usec
TE               298.4 K
D1          1.00000000 sec
TD0                      1

======== CHANNEL f1 ========
NUC1                    1H
P1              9.50 usec
PL1        0 dB
PL1W        8.16363049 W
SFO1      299.8718518 MHz

F2 - Processing parameters
SI                   32768
SF        299.8700037 MHz
WDW                     EM
SSB        0
LB                 0.30 Hz
GB         0
PC                   11.00
```

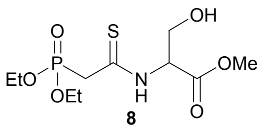

sp139-3
C13.ucl CDCl3 {C:\Bruker\TOPSPIN} jca 44

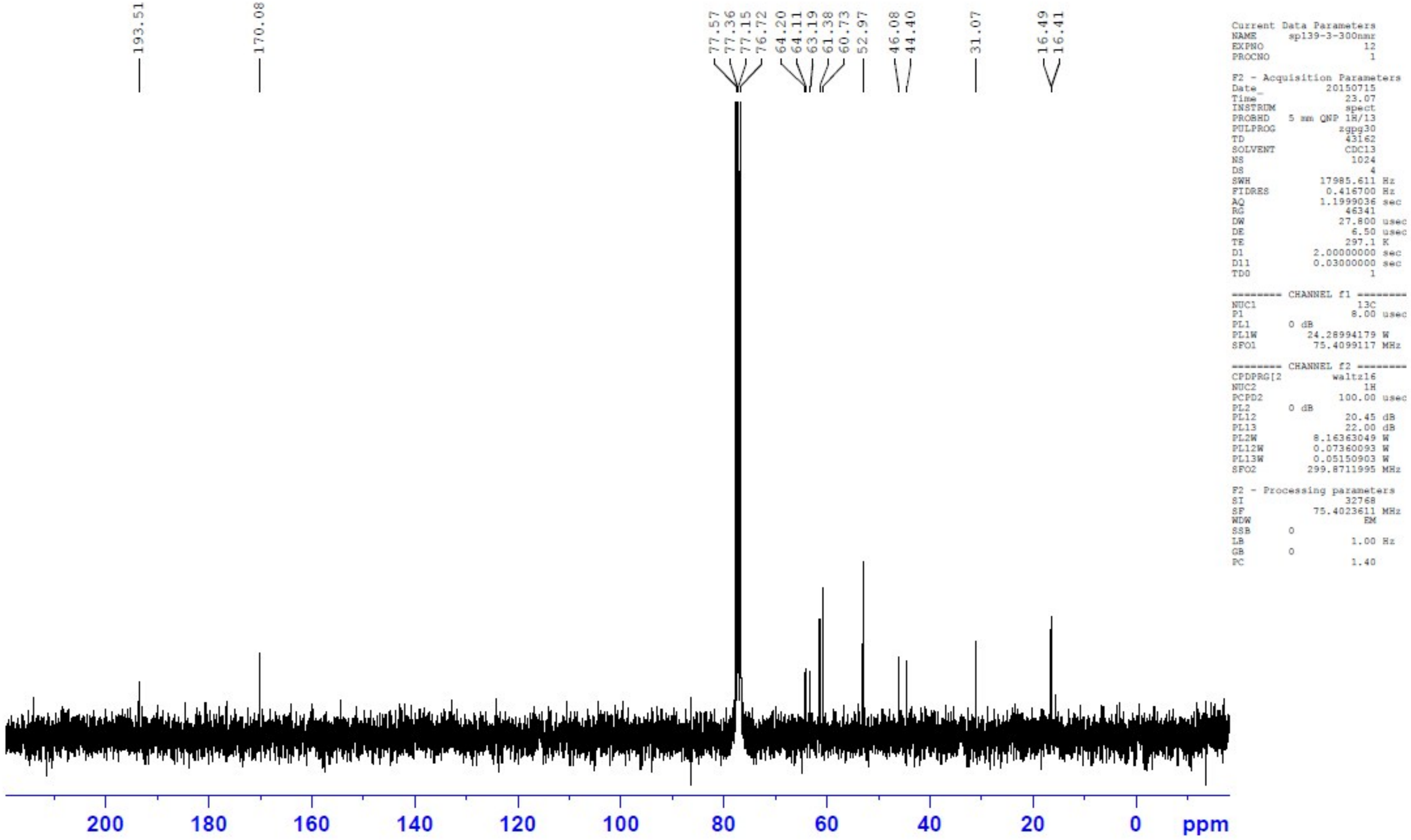

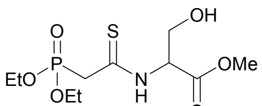

8

sp139-3
P31_with_proton_couplings CDCl3 {C:\Bruker\TOPSPIN} jca 46

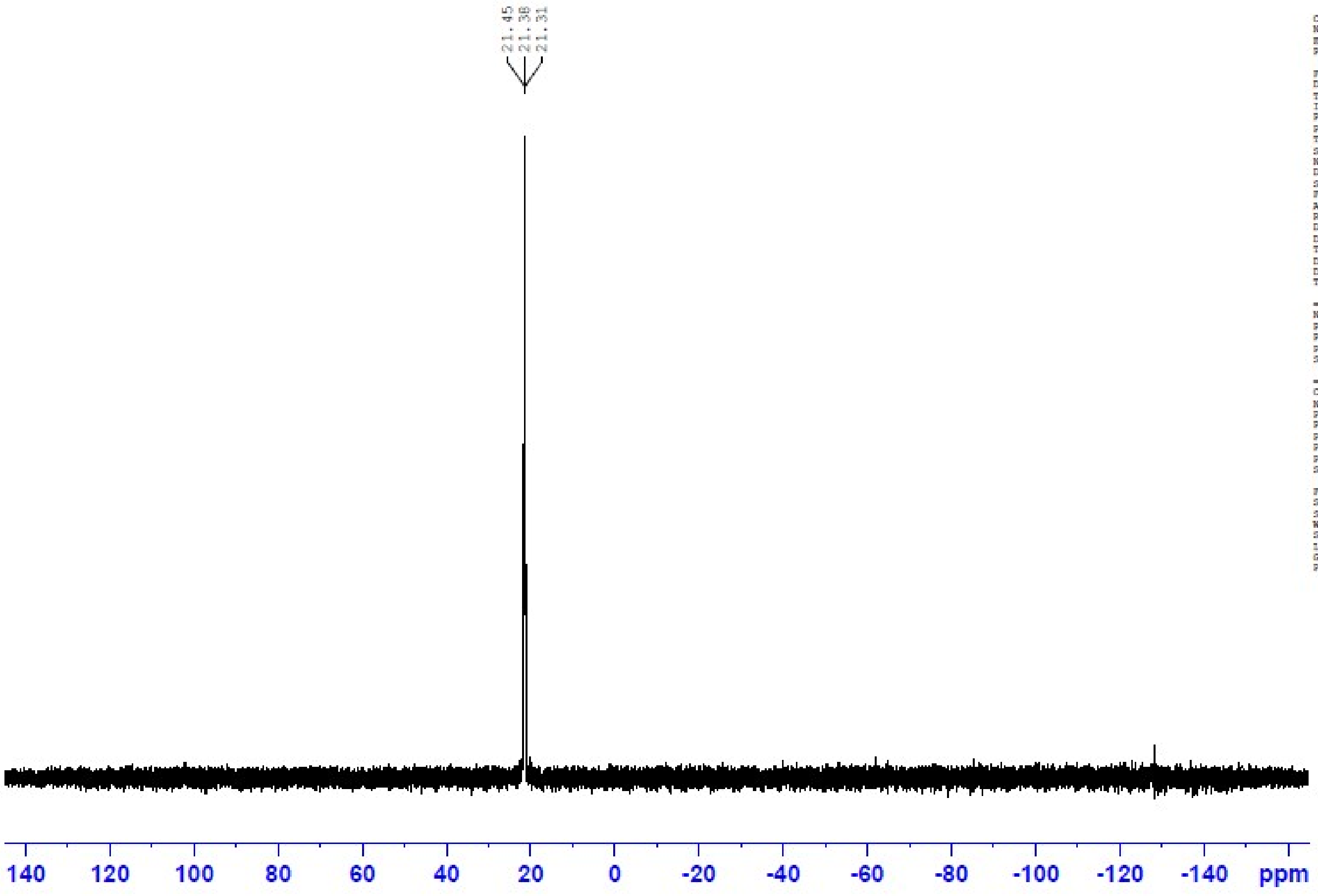


```
Current Data Parameters
NAME        sp139-3-P31coupled
EXPNO                     10
PROCNO                     1

F2 - Acquisition Parameters
Date_               20150721
Time                   11.50
INSTRUM                spect
PROBHD   5 mm QNP 1H/13
PULPROG              zggd30
TD                     65536
SOLVENT                CDCl3
NS                       256
DS                         4
SWH               37593.984 Hz
FIDRES             0.573639 Hz
AQ                0.8716288 sec
RG                     46341
DW                   13.300 usec
DE                     6.50 usec
TE                    299.6 K
D1               1.10000002 sec
D11              0.03000000 sec
TD0                        1

======== CHANNEL f1 ========
NUC1                     31P
P1                     10.45 usec
PL1          0 dB
PL1W            27.12837410 W
SFO1           121.3883871 MHz

======== CHANNEL f2 ========
CPDPRG[2             waltz16
NUC2                      1H
PCPD2                 100.00 usec
PL2          0 dB
PL12                   18.56 dB
PL2W             8.16363049 W
PL12W            0.11373220 W
SFO2           299.8725939 MHz

F2 - Processing parameters
SI                    131072
SF             121.3896010 MHz
WDW                       EM
SSB          0
LB                      1.00 Hz
GB           0
PC                      1.40
```

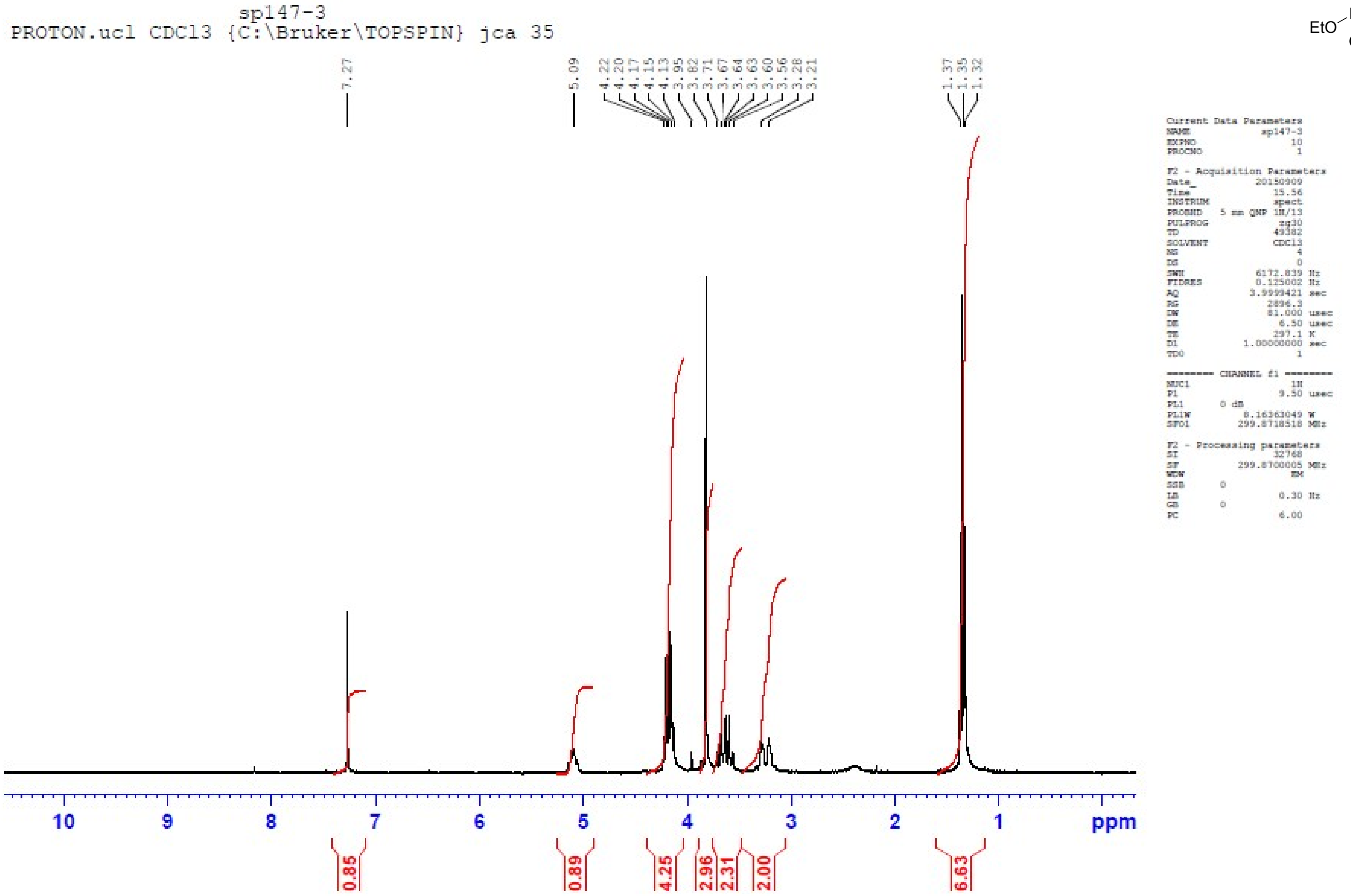

sp147-3
PROTON.ucl CDCl3 {C:\Bruker\TOPSPIN} jca 35
7.27
5.09
4.22
4.20
4.17
4.15
4.13
3.95
3.82
3.71
3.67
3.64
3.63
3.60
3.56
3.28
3.21
1.37
1.35
1.32
10
9
8
7
6
5
4
3
2
1
ppm
0.85
0.89
4.25
2.96
2.31
2.00
6.63
Current Data Parameters
NAME sp147-3
EXPNO 10
PROCNO 1
F2 - Acquisition Parameters
Date_ 20150909
Time 15.56
INSTRUM spect
PROBHD 5 mm QNP 1H/13
PULPROG zg30
TD 49382
SOLVENT CDCl3
NS 4
DS 0
SWH 6172.839 Hz
FIDRES 0.125002 Hz
AQ 3.9999421 sec
RG 2896.3
DW 81.000 usec
DE 6.50 usec
TE 297.1 K
D1 1.00000000 sec
TD0 1
======== CHANNEL f1 ========
NUC1 1H
P1 9.50 usec
PL1 0 dB
PL1W 8.16363049 W
SFO1 299.8718518 MHz
F2 - Processing parameters
SI 32768
SF 299.8700005 MHz
WDW EM
SSB 0
LB 0.30 Hz
GB 0
PC 6.00

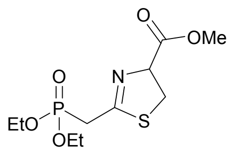

O
OMe
O
N
P
EtO
OEt
S

9

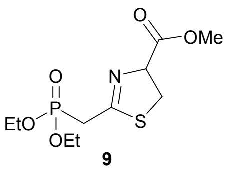

9

sp147-3-full data
C13_DayTime.ucl CDCl3 {W:\600} jca 40

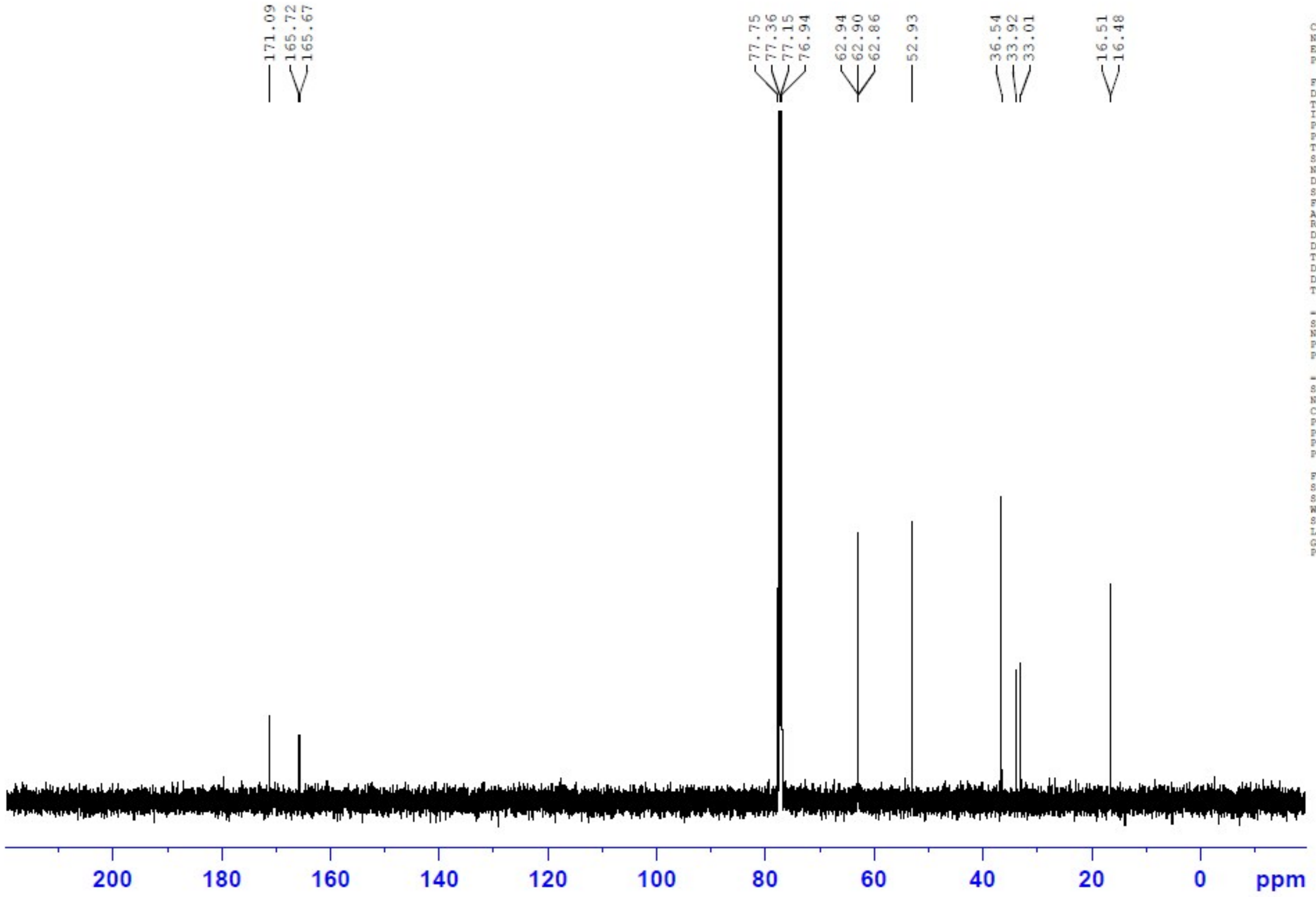


```
Current Data Parameters
NAME      sp147-3-full data
EXPNO                    13
PROCNO                    1

F2 - Acquisition Parameters
Date_              20160415
Time                  11.55
INSTRUM               AV600
PROBHD   5 mm CPDCH 13C
PULPROG              zgpg30
TD                    71914
SOLVENT               CDCl3
NS                       32
DS                        4
SWH               36057.691 Hz
FIDRES             0.501400 Hz
AQ                0.9972075 sec
RG                      203
DW                   13.867 usec
DE                    19.08 usec
TE                    298.0 K
D1               1.79999995 sec
D11              0.03000000 sec
TD0                       2

======== CHANNEL f1 ========
SFO1            150.9178981 MHz
NUC1                    13C
P1                     9.80 usec
PLW1            26.76899910 W

======== CHANNEL f2 ========
SFO2            600.1324005 MHz
NUC2                     1H
CPDPRG[2            waltz16
PCPD2                 70.00 usec
PLW2            13.76700020 W
PLW12            0.36513001 W
PLW13            0.17892000 W

F2 - Processing parameters
SI                    65536
SF              150.9027930 MHz
WDW                      EM
SSB      0
LB                     1.00 Hz
GB       0
PC                     1.40
```

sp147-3P31
P31.ucl CDCl3 {C:\Bruker\TOPSPIN} jca 35

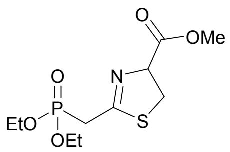

11

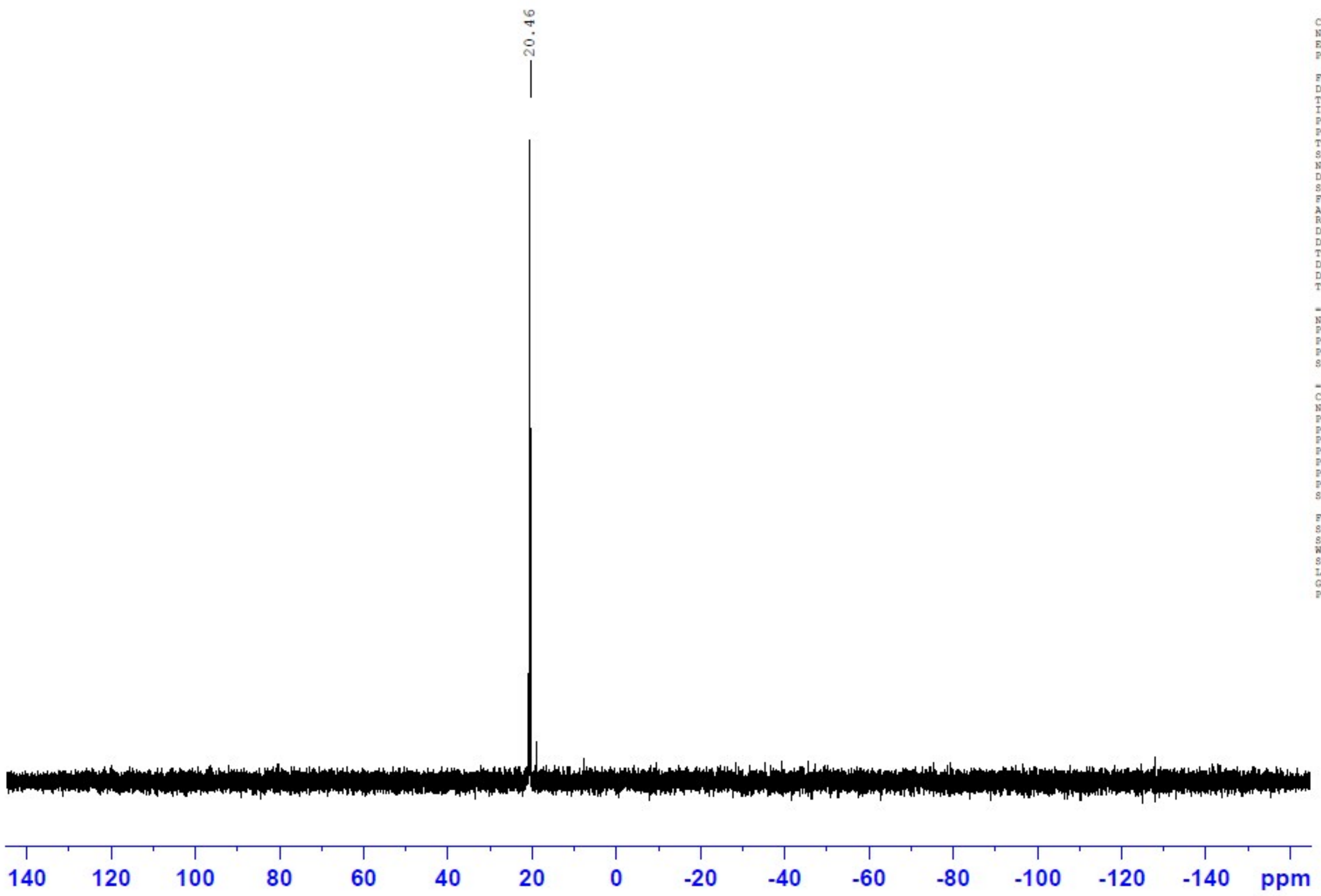


```
Current Data Parameters
NAME          sp147-3P31
EXPNO                 10
PROCNO                 1

F2 - Acquisition Parameters
Date_           20150909
Time               16.05
INSTRUM            spect
PROBHD   5 mm QNP 1H/13
PULPROG           zgpg30
TD                 58818
SOLVENT            CDCl3
NS                    64
DS                     4
SWH            37593.984 Hz
FIDRES          0.639158 Hz
AQ             0.7822794 sec
RG                 46341
DW                13.300 usec
DE                  6.50 usec
TE                 298.4 K
D1            1.29999995 sec
D11           0.03000000 sec
TD0                    1

======== CHANNEL f1 ========
NUC1                 31P
P1                 10.45 usec
PL1         0 dB
PL1W         27.12837410 W
SFO1         121.3883871 MHz

======== CHANNEL f2 ========
CPDPRG[2         waltz16
NUC2                  1H
PCPD2             100.00 usec
PL2         0 dB
PL12               18.56 dB
PL13               22.00 dB
PL2W          8.16363049 W
PL12W         0.11373220 W
PL13W         0.05150903 W
SFO2         299.8711995 MHz

F2 - Processing parameters
SI                131072
SF           121.3896010 MHz
WDW                   EM
SSB         0
LB                  1.00 Hz
GB          0
PC                  1.40
```

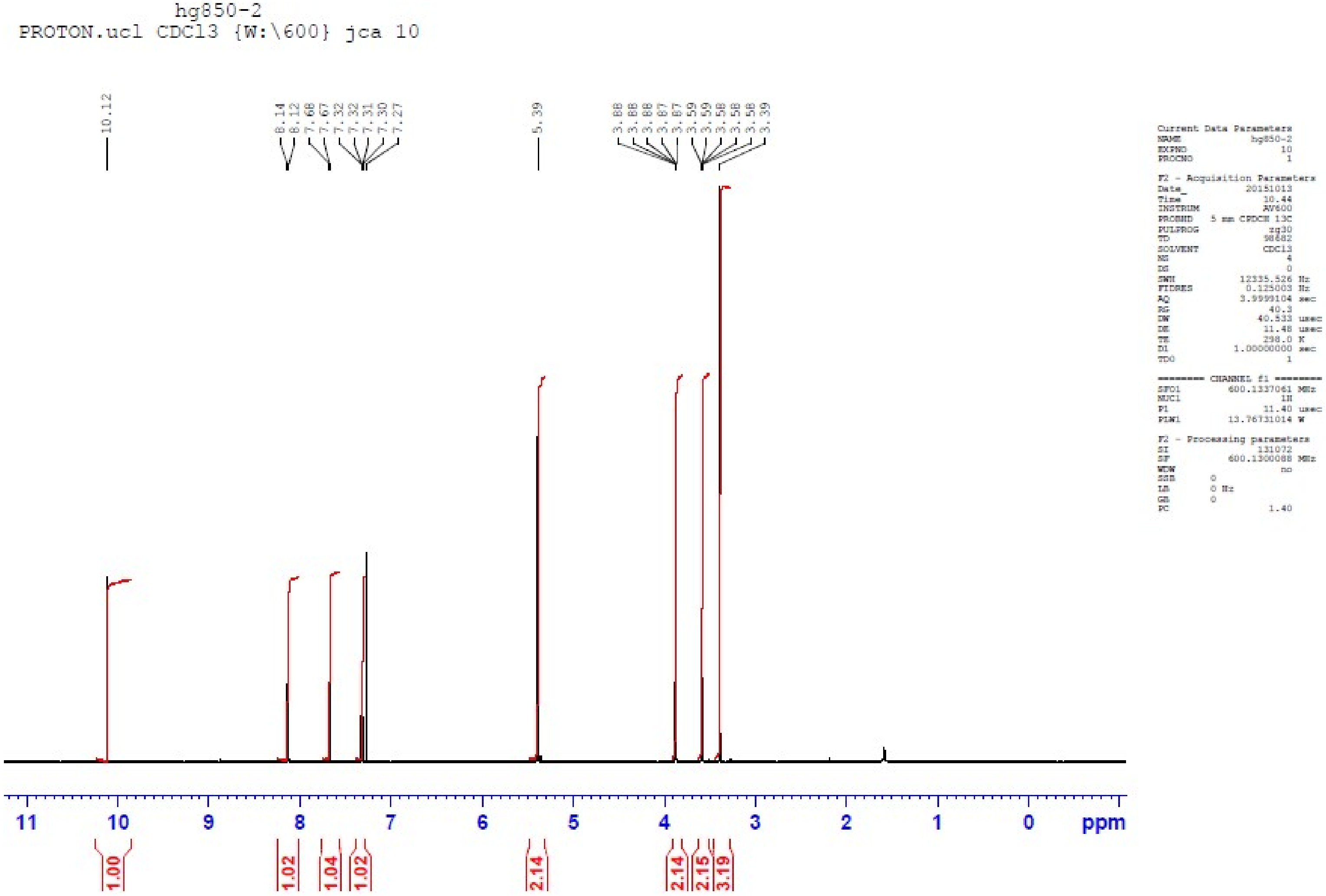

hg850-2
PROTON.ucl CDCl3 {W:\600} jca 10
10.12
8.14
8.12
7.68
7.67
7.32
7.32
7.31
7.30
7.27
5.39
3.88
3.88
3.88
3.87
3.87
3.59
3.59
3.58
3.58
3.58
3.39
11
10
9
8
7
6
5
4
3
2
1
0
ppm
1.00
1.02
1.04
1.02
2.14
2.14
2.15
3.19
Current Data Parameters
NAME hg850-2
EXPNO 10
PROCNO 1
F2 - Acquisition Parameters
Date_ 20151013
Time 10.44
INSTRUM AV600
PROBHD 5 mm CPDCH 13C
PULPROG zg30
TD 98682
SOLVENT CDCl3
NS 4
DS 0
SWH 12335.526 Hz
FIDRES 0.125003 Hz
AQ 3.9999104 sec
RG 40.3
DW 40.533 usec
DE 11.48 usec
TE 298.0 K
D1 1.00000000 sec
TD0 1
======== CHANNEL f1 ========
SFO1 600.1337061 MHz
NUC1 1H
P1 11.40 usec
PLW1 13.76731014 W
F2 - Processing parameters
SI 131072
SF 600.1300088 MHz
WDW no
SSB 0
LB 0 Hz
GB 0
PC 1.40

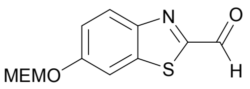

MEMO
N
S
O
H

10

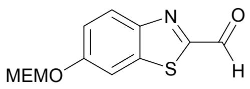

10

C13CPD.ucl CDCl3 {W:\600} tds 10

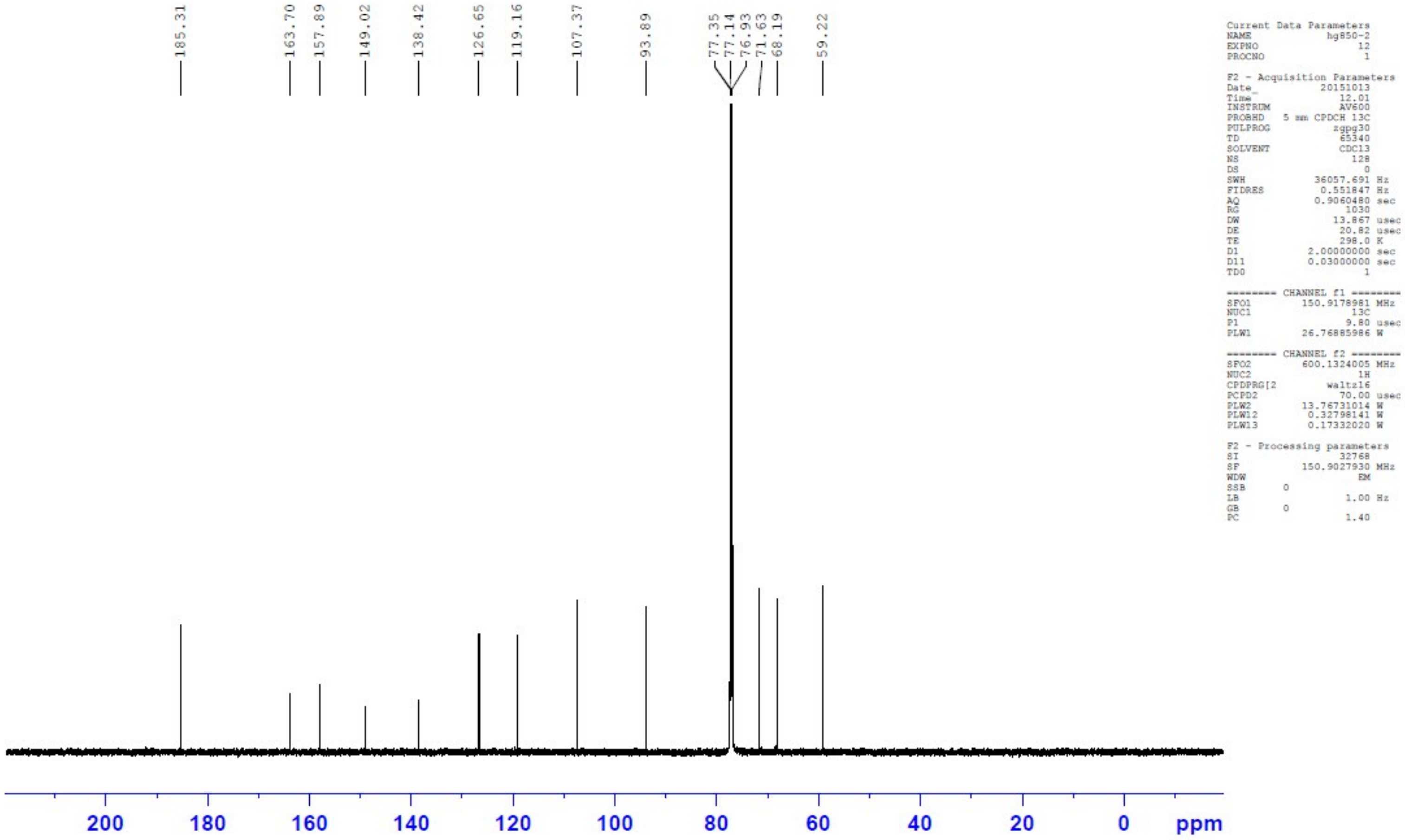

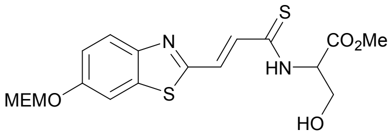
S
N
CO2Me
HN
MEMO
S
HO

11

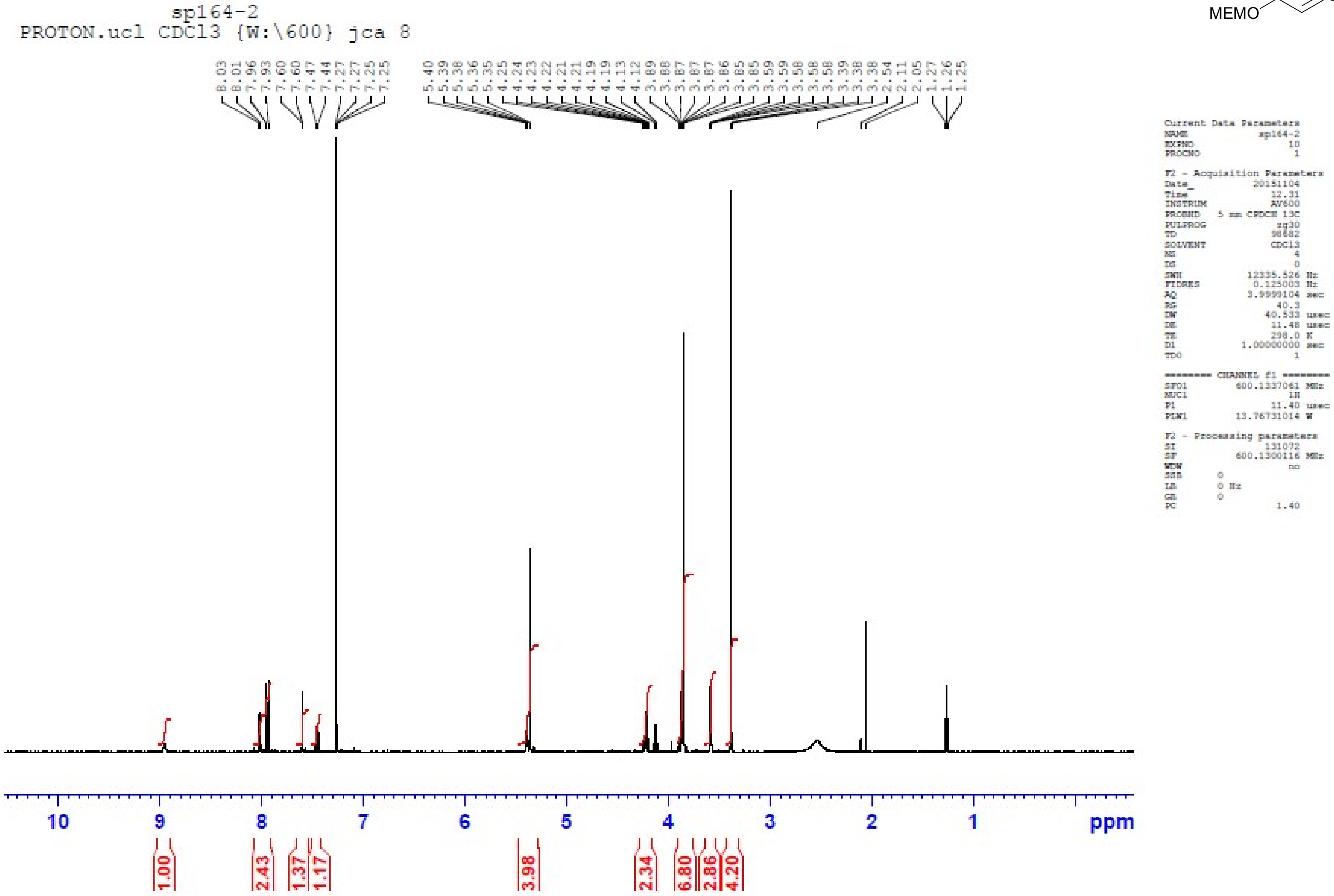
sp164-2
PROTON.ucl CDCl3 {W:\600} jca 8
10
9
8
7
6
5
4
3
2
1
ppm
1.00
2.43
1.37
1.17
3.98
2.34
6.80
2.86
4.20

sp164-2
C13_DayTime.ucl CDCl3 {W:\600} jca 8

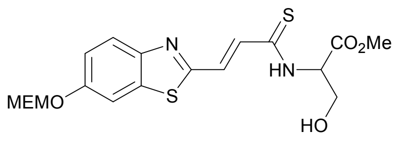

11

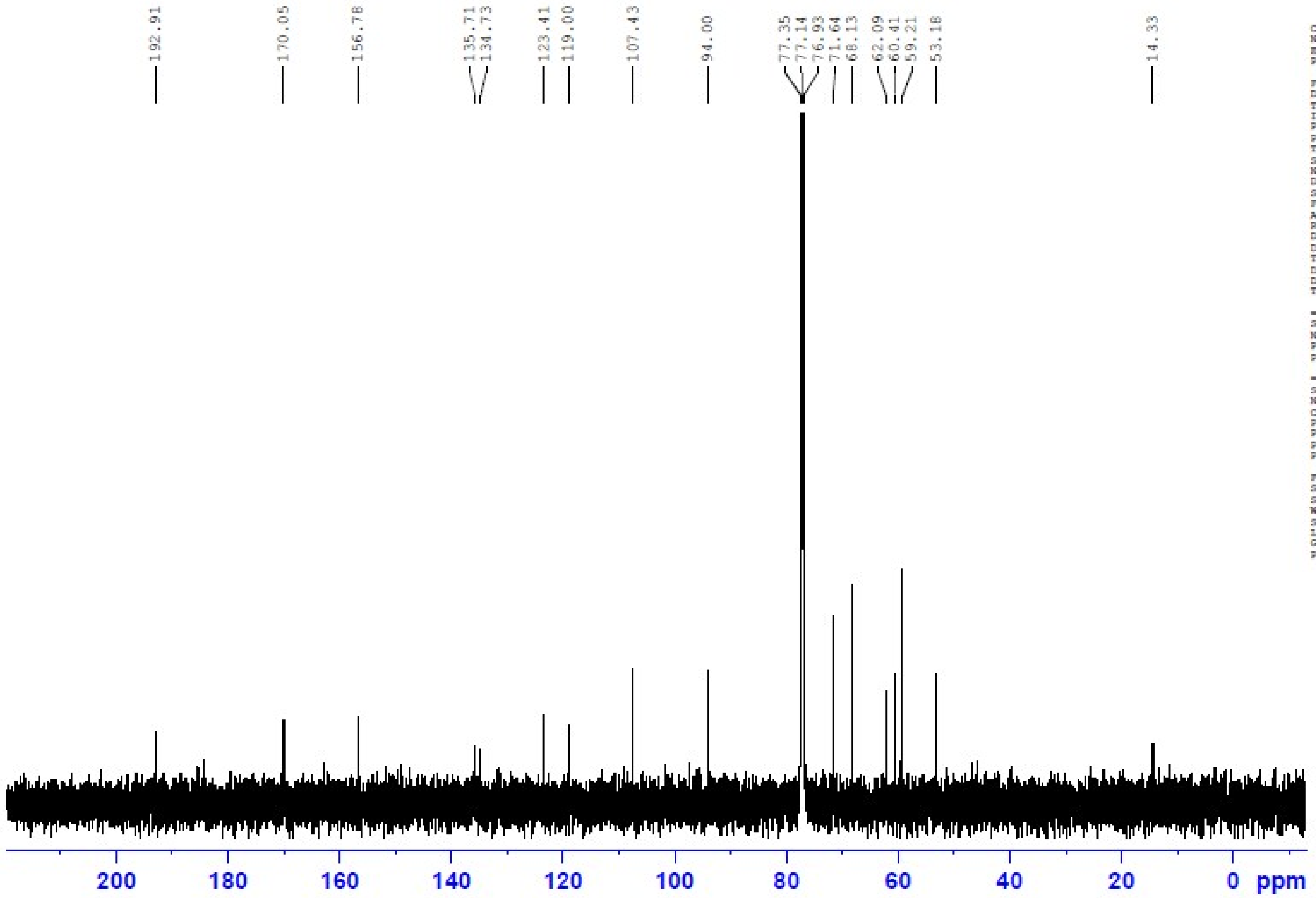


```
Current Data Parameters
NAME              sp164-2
EXPNO                  11
PROCNO                  1

F2 - Acquisition Parameters
Date_            20151104
Time                12.34
INSTRUM             AV600
PROBHD   5 mm CPDCH 13C
PULPROG           zgpg30
TD                 65340
SOLVENT            CDCl3
NS                    32
DS                     0
SWH            36057.691 Hz
FIDRES          0.551847 Hz
AQ             0.9060480 sec
RG                  1030
DW                13.867 usec
DE                 20.82 usec
TE                 298.0 K
D1            2.00000000 sec
D11           0.03000000 sec
TD0                    1

======== CHANNEL f1 ========
SFO1         150.9178981 MHz
NUC1                 13C
P1                  9.80 usec
PLW1         26.76885986 W

======== CHANNEL f2 ========
SFO2         600.1324005 MHz
NUC2                  1H
CPDPRG[2         waltz16
PCPD2              70.00 usec
PLW2         13.76731014 W
PLW12         0.32798141 W
PLW13         0.17332020 W

F2 - Processing parameters
SI                 32768
SF           150.9027930 MHz
WDW                   EM
SSB      0
LB                  1.00 Hz
GB       0
PC                  1.40
```

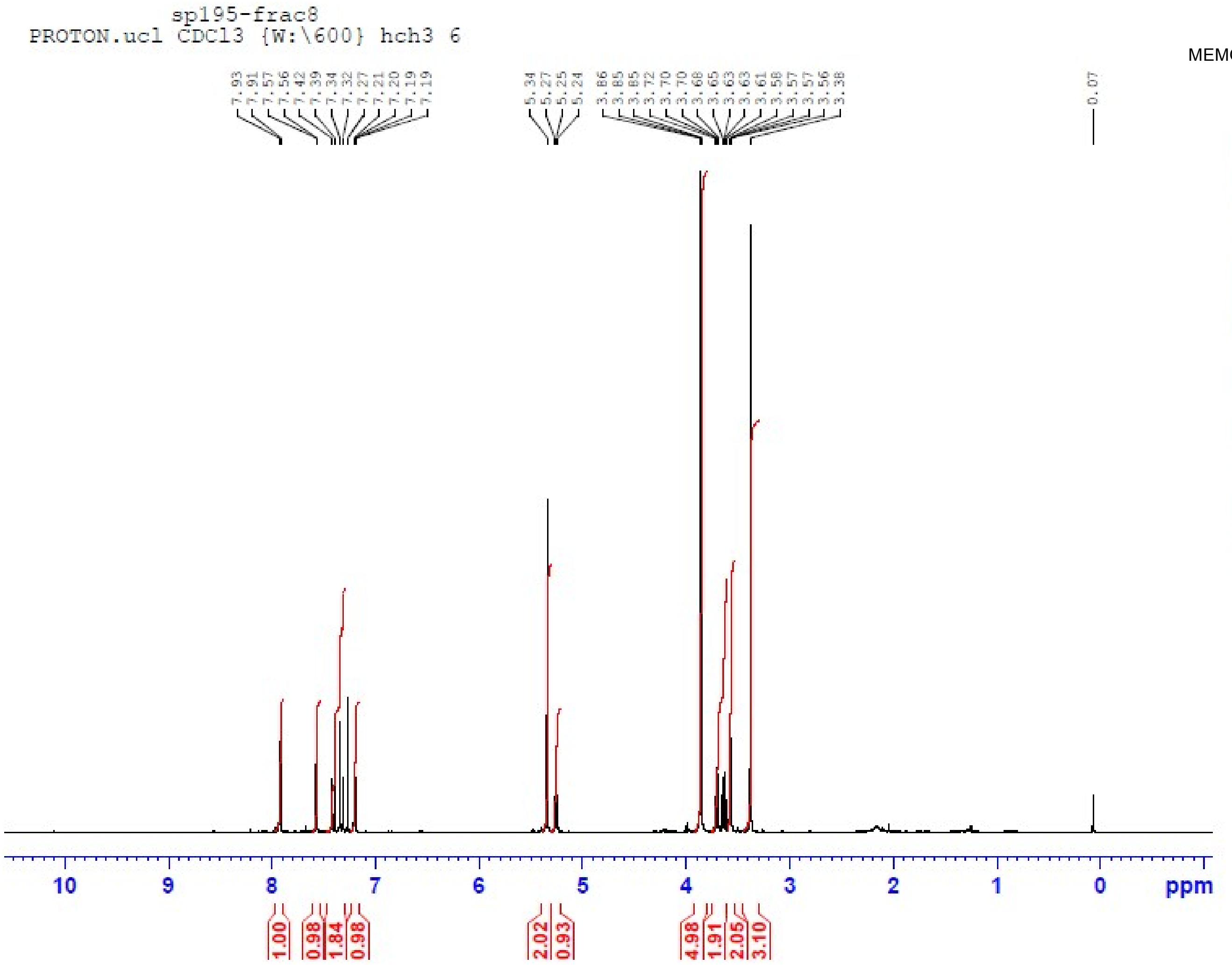

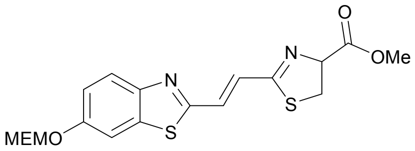

12

Current Data Parameters
NAME sp195-frac8
EXPNO 10
PROCNO 1

F2 - Acquisition Parameters
Date_ 20160122
Time 14.57
INSTRUM AV600
PROBHD 5 mm CPDCH 13C
PULPROG zg30
TD 98682
SOLVENT CDCl3
NS 4
DS 0
SWH 12335.526 Hz
FIDRES 0.125003 Hz
AQ 3.9999104 sec
RG 40.3
DW 40.533 usec
DE 11.48 usec
TE 298.0 K
D1 1.00000000 sec
TD0 1

======== CHANNEL f1 ========
SFO1 600.1337061 MHz
NUC1 1H
P1 11.40 usec
PLW1 13.76731014 W

F2 - Processing parameters
SI 131072
SF 600.1300116 MHz
WDW no
SSB 0
LB 0 Hz
GB 0
PC 1.40

sp195-frac8
C13_DayTime.ucl CDCl3 {W:\600} hch3 6

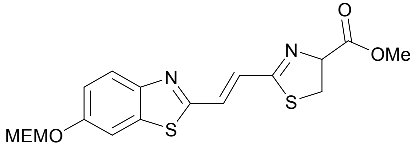

12

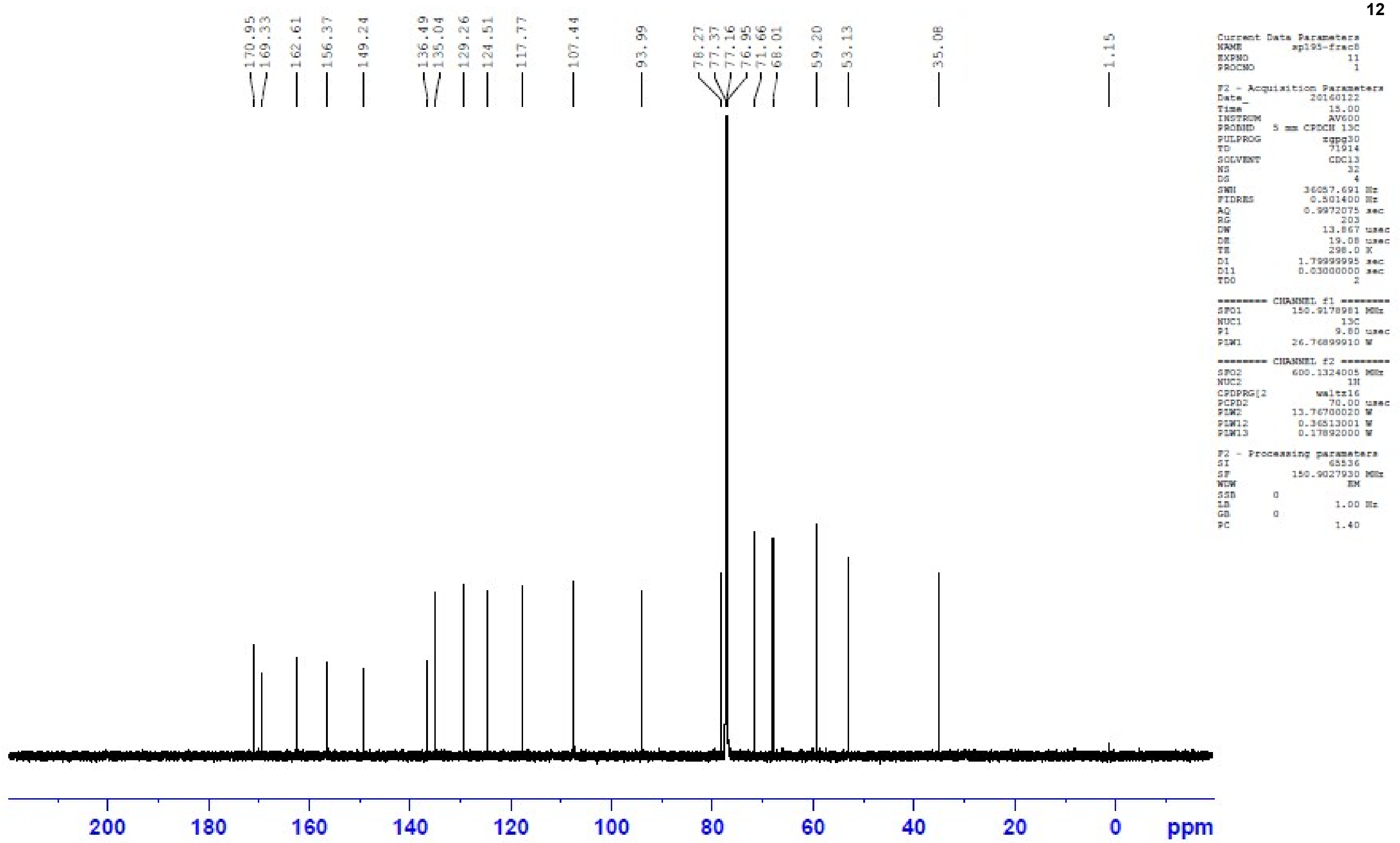

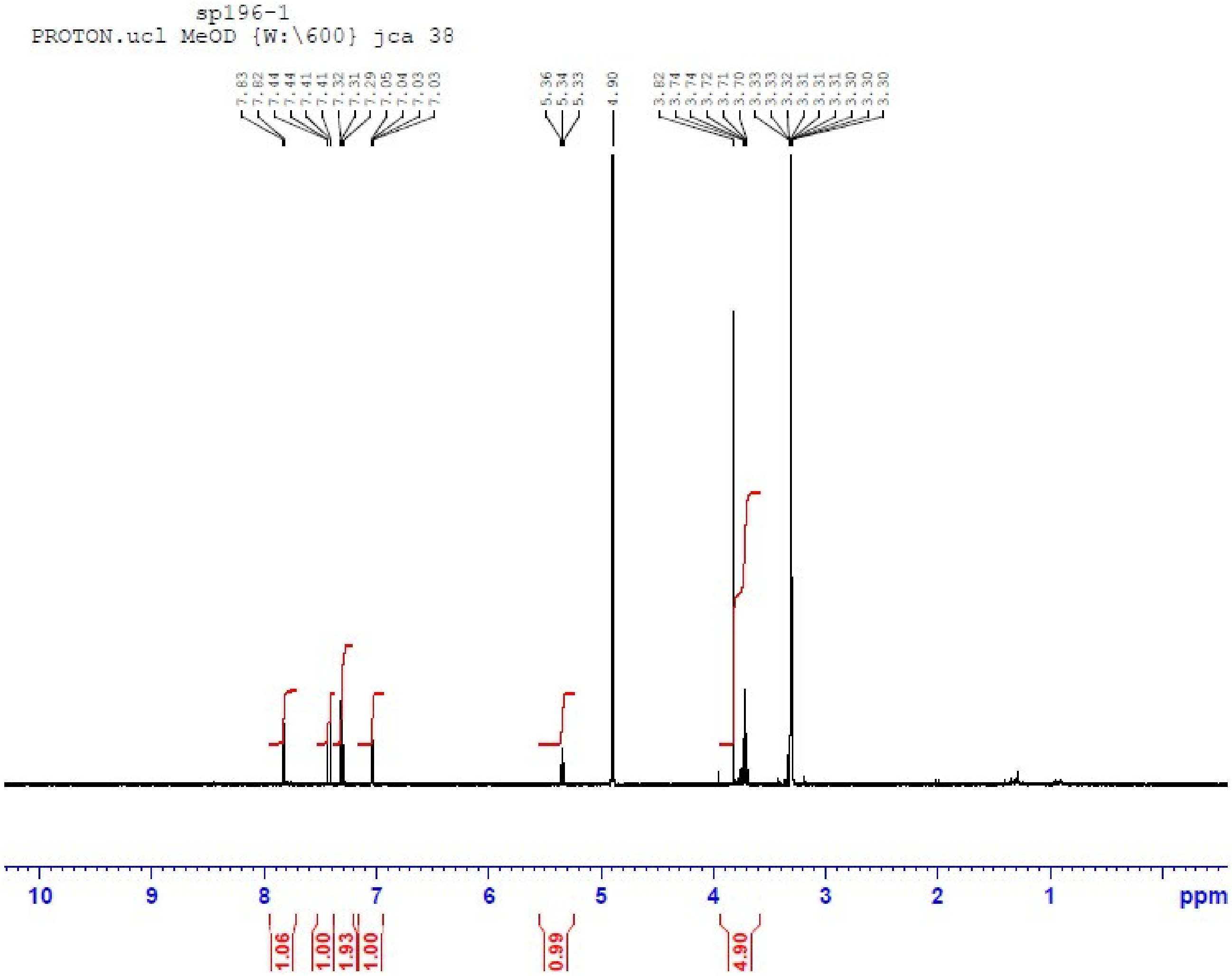

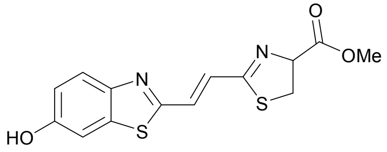

6

```
Current Data Parameters
NAME              sp196-1
EXPNO                  10
PROCNO                  1

F2 - Acquisition Parameters
Date_            20160126
Time                13.28
INSTRUM             AV600
PROBHD   5 mm CPDCH 13C
PULPROG              zg30
TD                  98682
SOLVENT              MeOD
NS                      4
DS                      0
SWH             12335.526 Hz
FIDRES           0.125003 Hz
AQ              3.9999104 sec
RG                   40.3
DW                 40.533 usec
DE                  11.48 usec
TE                  298.0 K
D1             1.00000000 sec
TD0                     1

======== CHANNEL f1 ========
SFO1          600.1337061 MHz
NUC1                   1H
P1                  11.40 usec
PLW1          13.76731014 W

F2 - Processing parameters
SI                 131072
SF            600.1300141 MHz
WDW                    no
SSB       0
LB        0 Hz
GB        0
PC                   1.40
```

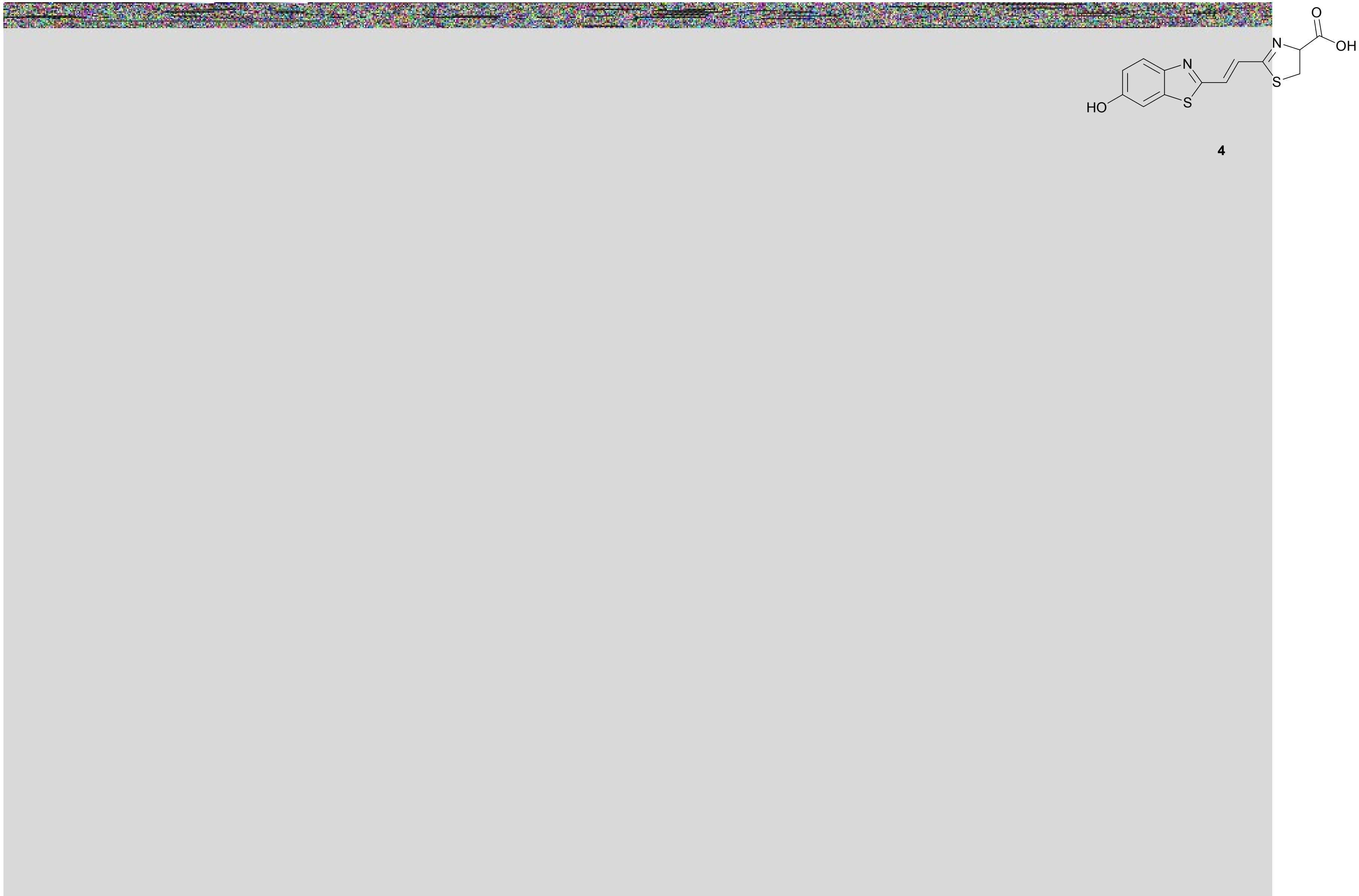

4

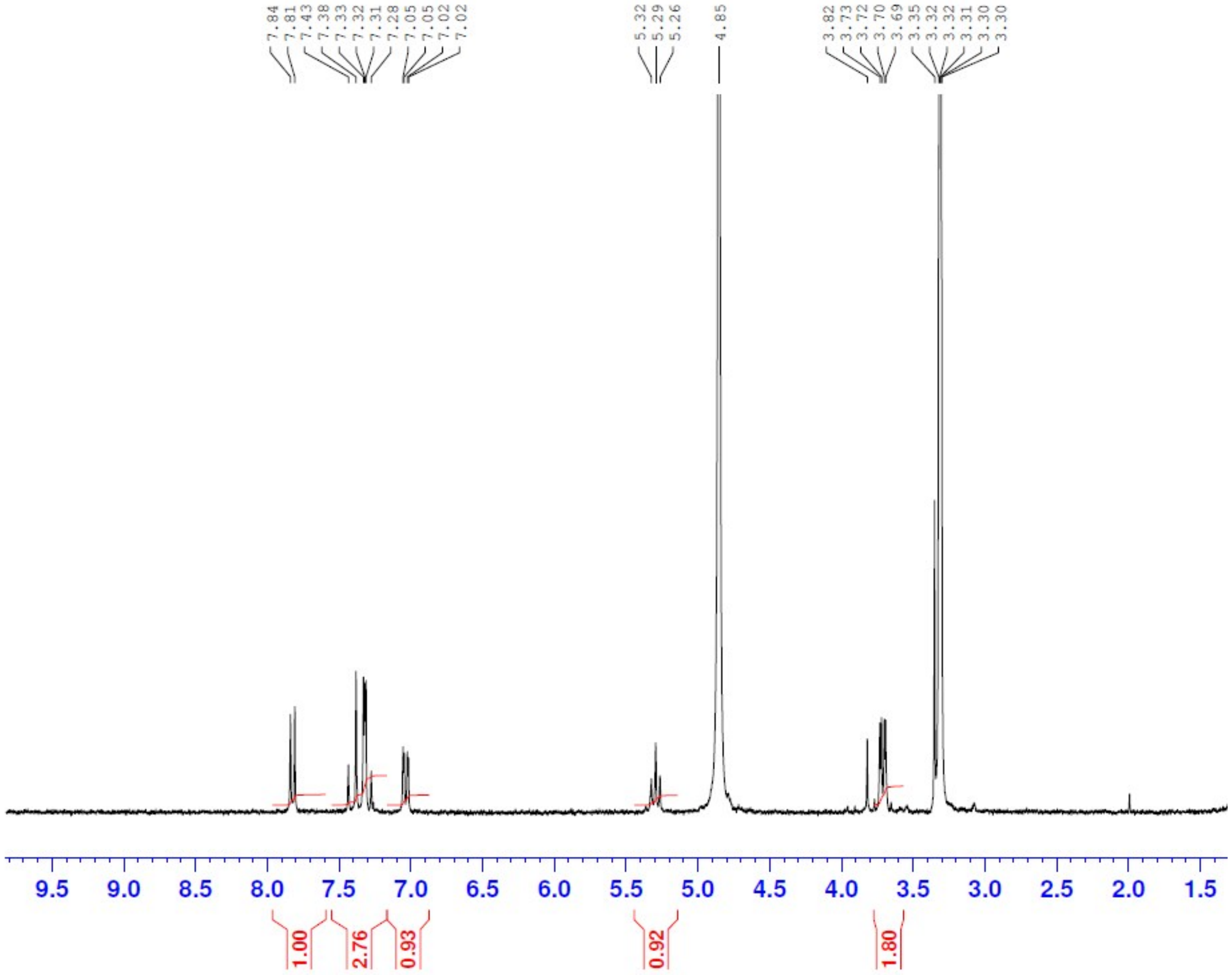

7.84
7.81
7.43
7.38
7.33
7.32
7.31
7.28
7.05
7.05
7.02
7.02
5.32
5.29
5.26
4.85
3.82
3.73
3.72
3.70
3.69
3.35
3.32
3.32
3.31
3.30
3.30
9.5
9.0
8.5
8.0
7.5
7.0
6.5
6.0
5.5
5.0
4.5
4.0
3.5
3.0
2.5
2.0
1.5
1.00
2.76
0.93
0.92
1.80

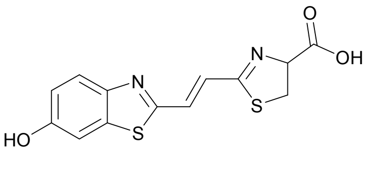

HO
N
S
N
S
O
OH

4

sp215
C13_DayTime.ucl MeOD {W:\600} jca 26

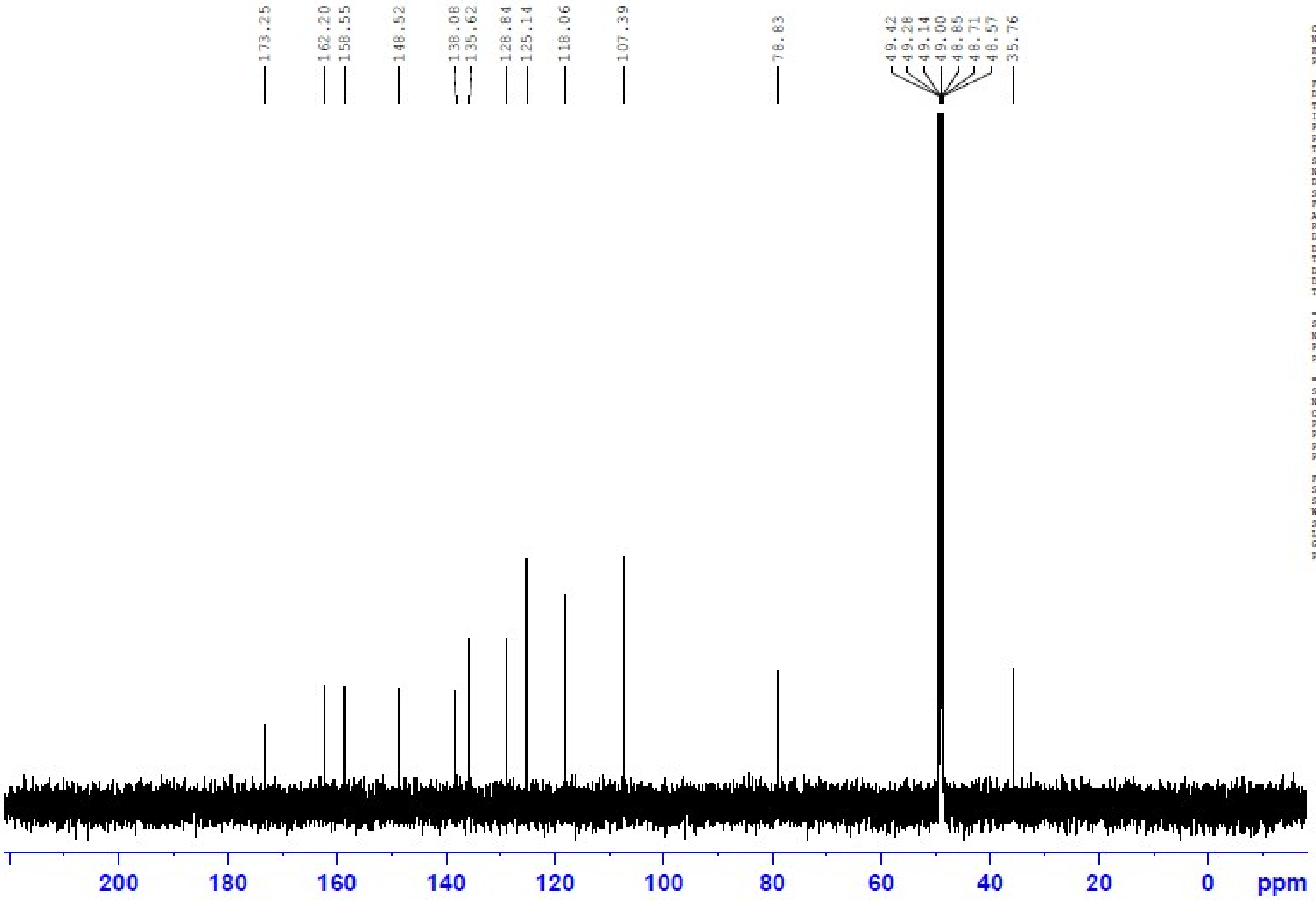

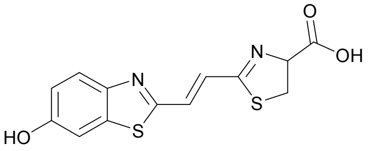

4

```
Current Data Parameters
NAME       sp215-full data
EXPNO                   12
PROCNO                   1

F2 - Acquisition Parameters
Date_             20160420
Time                 14.48
INSTRUM              AV600
PROBHD   5 mm CPDCH 13C
PULPROG            zgpg30
TD                   71914
SOLVENT               MeOD
NS                      32
DS                       4
SWH             36057.691 Hz
FIDRES           0.501400 Hz
AQ              0.9972075 sec
RG                     203
DW                  13.867 usec
DE                   19.08 usec
TE                   298.0 K
D1              1.79999995 sec
D11             0.03000000 sec
TD0                      2

======== CHANNEL f1 ========
SFO1          150.9178981 MHz
NUC1                   13C
P1                    9.80 usec
PLW1           26.76899910 W

======== CHANNEL f2 ========
SFO2          600.1324005 MHz
NUC2                    1H
CPDPRG[2           waltz16
PCPD2                70.00 usec
PLW2           13.76700020 W
PLW12           0.36513001 W
PLW13           0.17892000 W

F2 - Processing parameters
SI                   65536
SF            150.9025980 MHz
WDW                     EM
SSB       0
LB                    1.00 Hz
GB        0
PC                    1.40
```

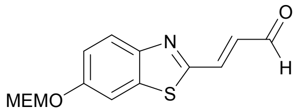

13

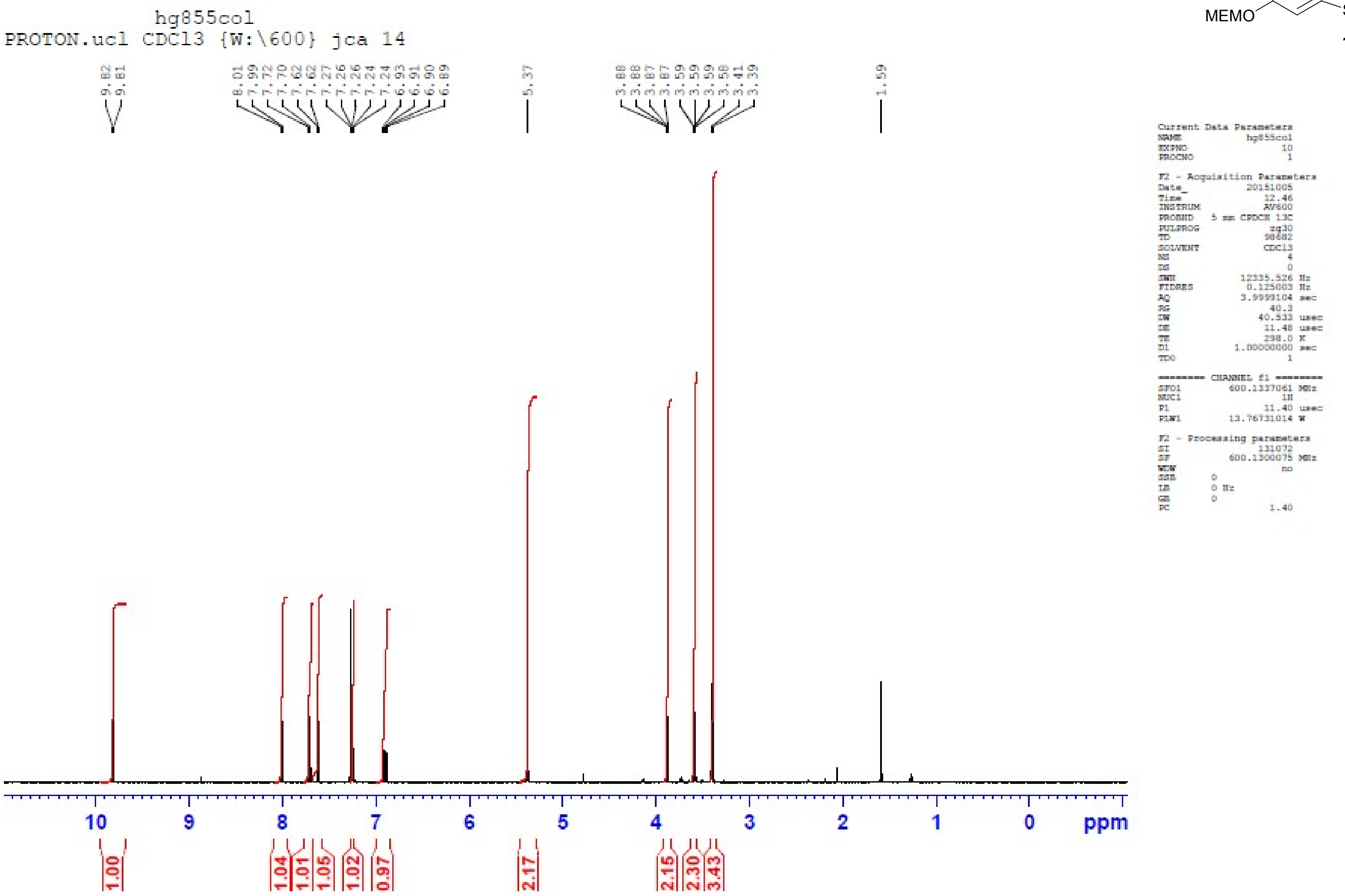

hg855col
PROTON.ucl CDCl3 {W:\600} jca 14
9.82
9.81
8.01
7.99
7.72
7.70
7.62
7.62
7.27
7.26
7.26
7.24
7.24
6.93
6.91
6.90
6.89
5.37
3.88
3.88
3.87
3.87
3.59
3.59
3.59
3.58
3.41
3.39
1.59
10
9
8
7
6
5
4
3
2
1
0
ppm
1.00
1.04
1.01
1.05
1.02
0.97
2.17
2.15
2.30
3.43
Current Data Parameters
NAME hg855col
EXPNO 10
PROCNO 1
F2 - Acquisition Parameters
Date_ 20151005
Time 12.46
INSTRUM AV600
PROBHD 5 mm CPDCH 13C
PULPROG zg30
TD 98682
SOLVENT CDCl3
NS 4
DS 0
SWH 12335.526 Hz
FIDRES 0.125003 Hz
AQ 3.9999104 sec
RG 40.3
DW 40.533 usec
DE 11.48 usec
TE 298.0 K
D1 1.00000000 sec
TD0 1
======== CHANNEL f1 ========
SFO1 600.1337061 MHz
NUC1 1H
P1 11.40 usec
PLW1 13.76731014 W
F2 - Processing parameters
SI 131072
SF 600.1300075 MHz
WDW no
SSB 0
LB 0 Hz
GB 0
PC 1.40

hg855col
C13CPD.ucl CDCl3 {W:\600} jca 14

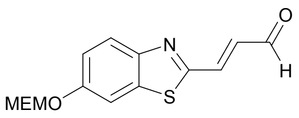

13

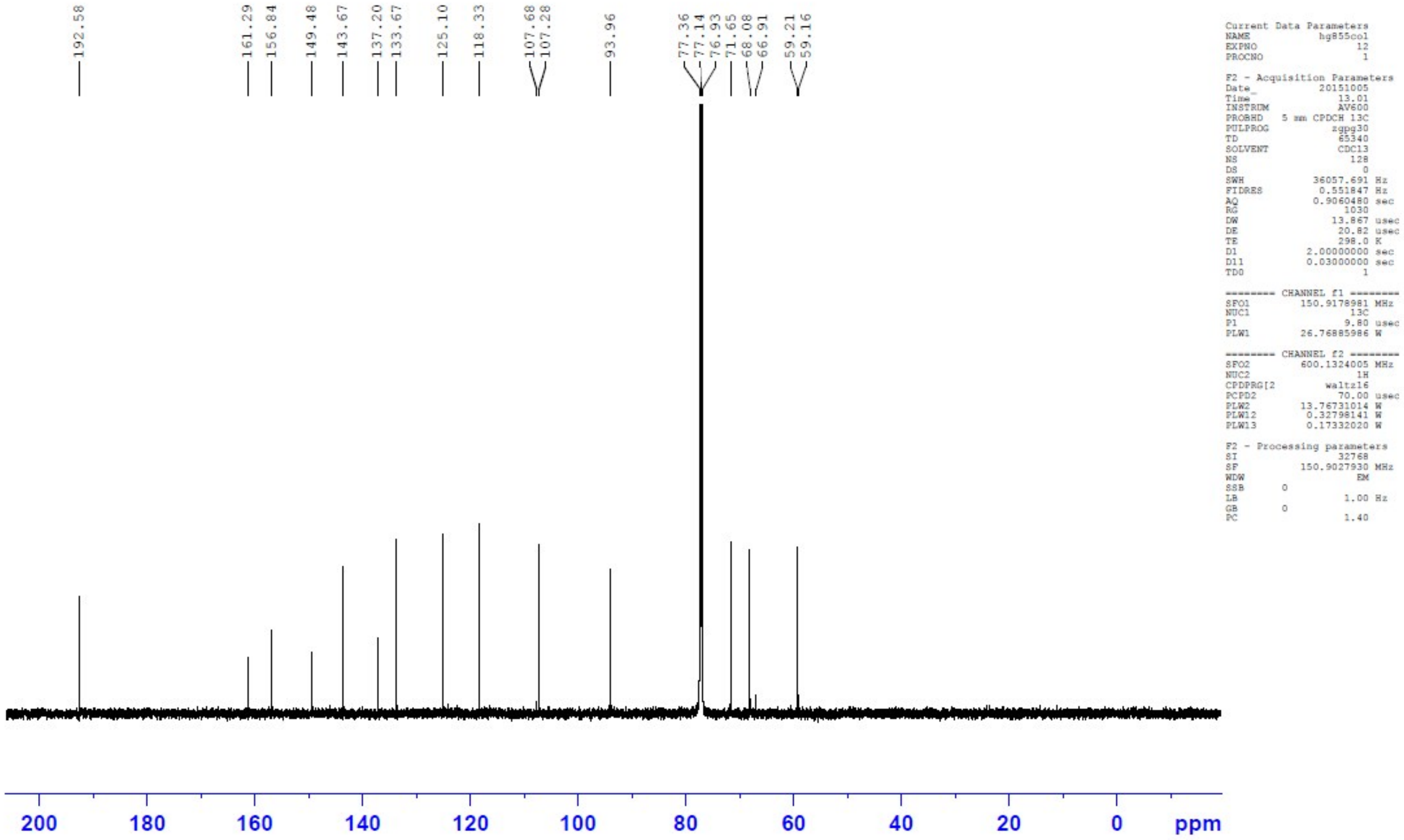


```
Current Data Parameters
NAME              hg855col
EXPNO                   12
PROCNO                   1

F2 - Acquisition Parameters
Date_             20151005
Time                 13.01
INSTRUM              AV600
PROBHD   5 mm CPDCH 13C
PULPROG            zgpg30
TD                   65340
SOLVENT              CDCl3
NS                     128
DS                       0
SWH              36057.691 Hz
FIDRES            0.551847 Hz
AQ               0.9060480 sec
RG                    1030
DW                  13.867 usec
DE                   20.82 usec
TE                   298.0 K
D1              2.00000000 sec
D11             0.03000000 sec
TD0                      1

======== CHANNEL f1 ========
SFO1           150.9178981 MHz
NUC1                   13C
P1                    9.80 usec
PLW1           26.76885986 W

======== CHANNEL f2 ========
SFO2           600.1324005 MHz
NUC2                    1H
CPDPRG[2           waltz16
PCPD2                70.00 usec
PLW2           13.76731014 W
PLW12           0.32798141 W
PLW13           0.17332020 W

F2 - Processing parameters
SI                   32768
SF             150.9027930 MHz
WDW                     EM
SSB      0
LB                    1.00 Hz
GB       0
PC                    1.40
```

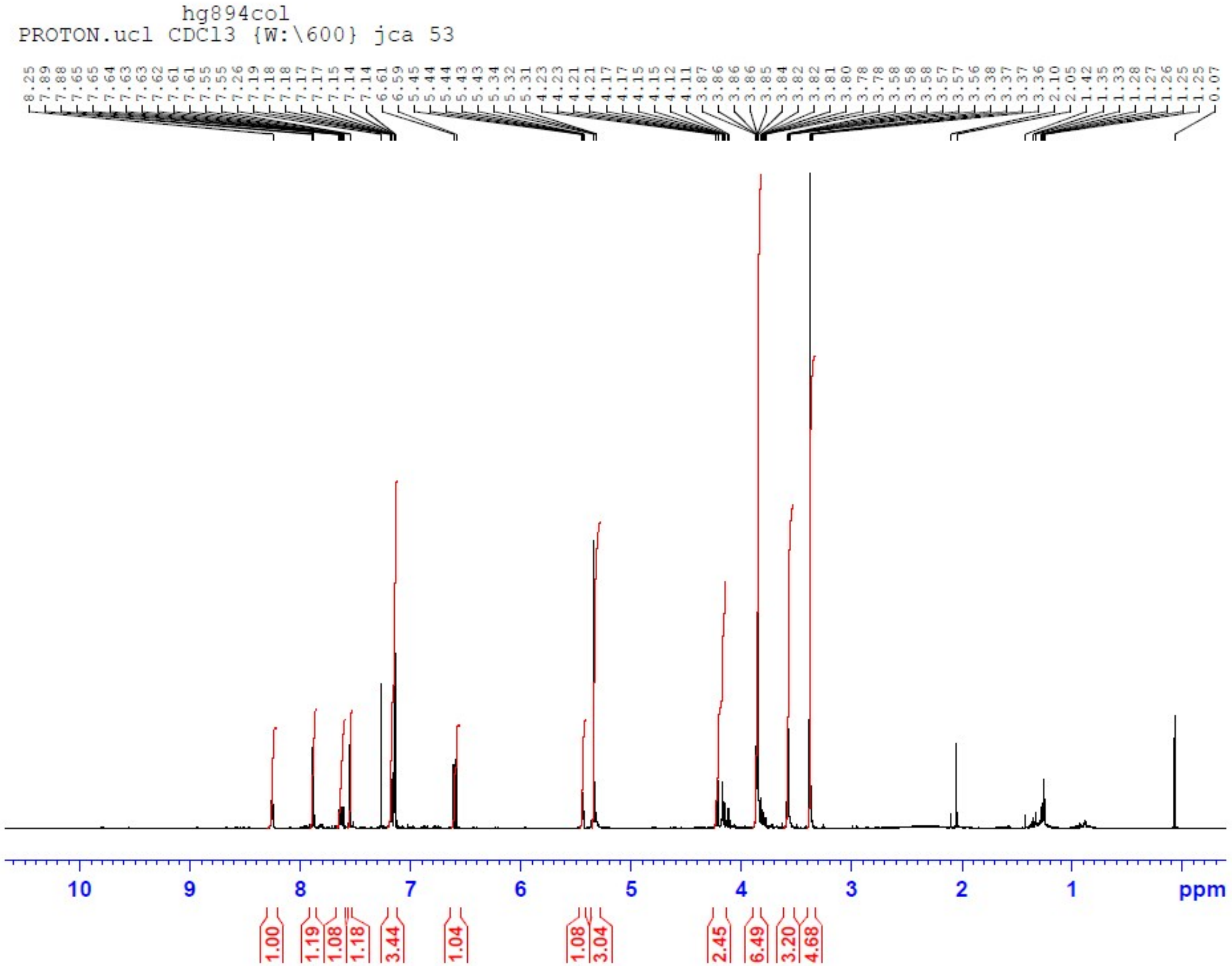

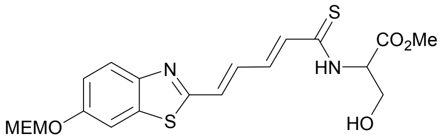

14

```
Current Data Parameters
NAME          hg894col
EXPNO               10
PROCNO               1

F2 - Acquisition Parameters
Date_         20160120
Time             12.53
INSTRUM          AV600
PROBHD   5 mm CPDCH 13C
PULPROG           zg30
TD               98682
SOLVENT          CDCl3
NS                   4
DS                   0
SWH          12335.526 Hz
FIDRES        0.125003 Hz
AQ           3.9999104 sec
RG                40.3
DW              40.533 usec
DE               11.48 usec
TE               298.0 K
D1          1.00000000 sec
TD0                  1

======== CHANNEL f1 ========
SFO1       600.1337061 MHz
NUC1                1H
P1               11.40 usec
PLW1       13.76731014 W

F2 - Processing parameters
SI              131072
SF         600.1300116 MHz
WDW                 no
SSB       0
LB        0 Hz
GB        0
PC                1.40
```

hg894col
C13_DayTime.ucl CDCl3 {W:\600} jca 53

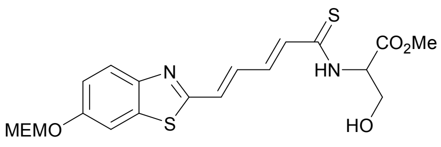

14

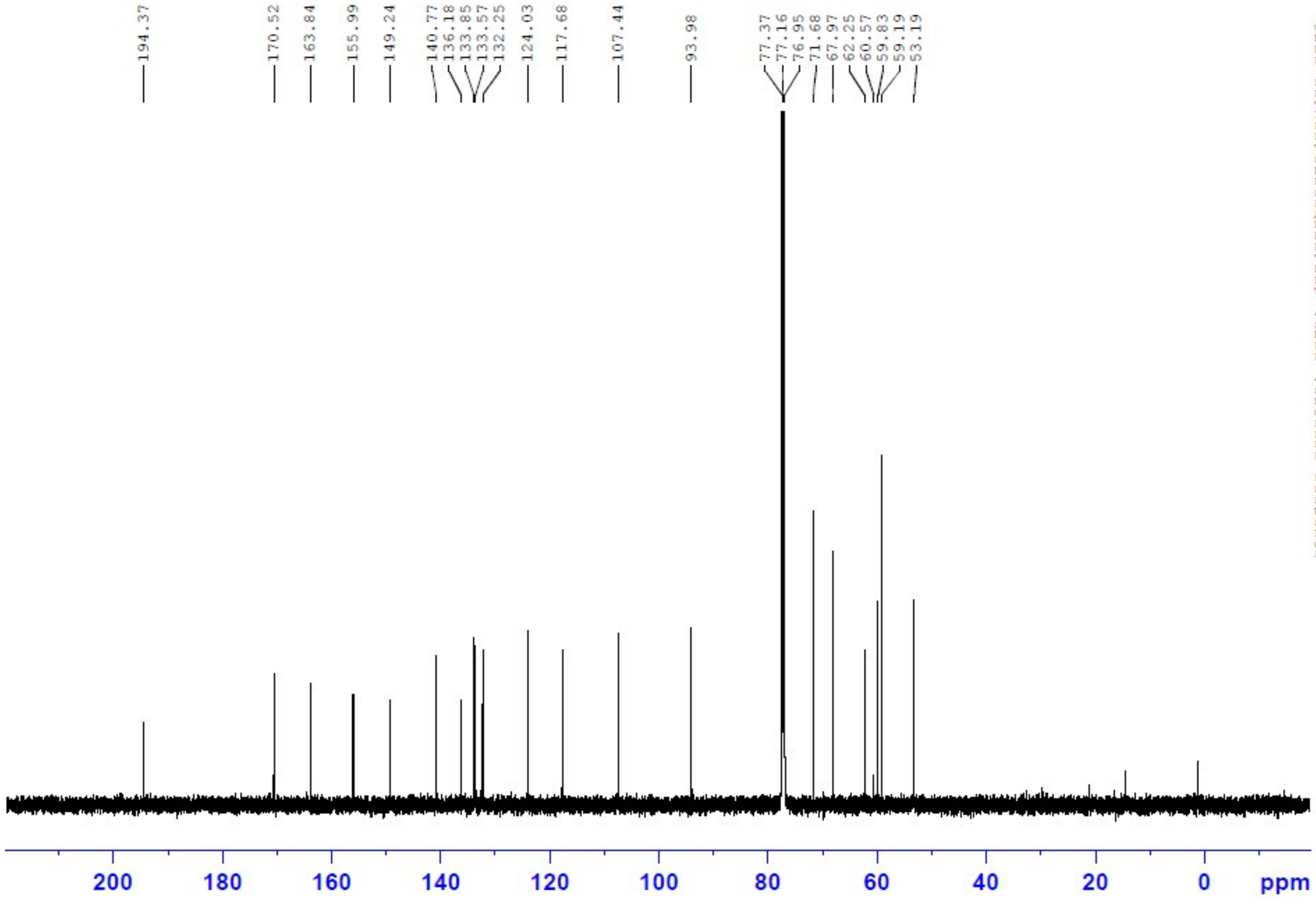


```
Current Data Parameters
NAME             hg894col
EXPNO                  11
PROCNO                  1

F2 - Acquisition Parameters
Date_            20160120
Time                12.56
INSTRUM             AV600
PROBHD   5 mm CPDCH 13C
PULPROG           zgpg30
TD                  71914
SOLVENT             CDCl3
NS                     32
DS                      4
SWH             36057.691 Hz
FIDRES           0.501400 Hz
AQ              0.9972075 sec
RG                    203
DW                 13.867 usec
DE                  19.08 usec
TE                  298.0 K
D1             1.79999995 sec
D11            0.03000000 sec
TD0                     2

======== CHANNEL f1 ========
SFO1          150.9178981 MHz
NUC1                  13C
P1                   9.80 usec
PLW1          26.76899910 W

======== CHANNEL f2 ========
SFO2          600.1324005 MHz
NUC2                   1H
CPDPRG[2          waltz16
PCPD2               70.00 usec
PLW2          13.76700020 W
PLW12          0.36513001 W
PLW13          0.17892000 W

F2 - Processing parameters
SI                  65536
SF            150.9027930 MHz
WDW                    EM
SSB       0
LB                   1.00 Hz
GB        0
PC                   1.40
```

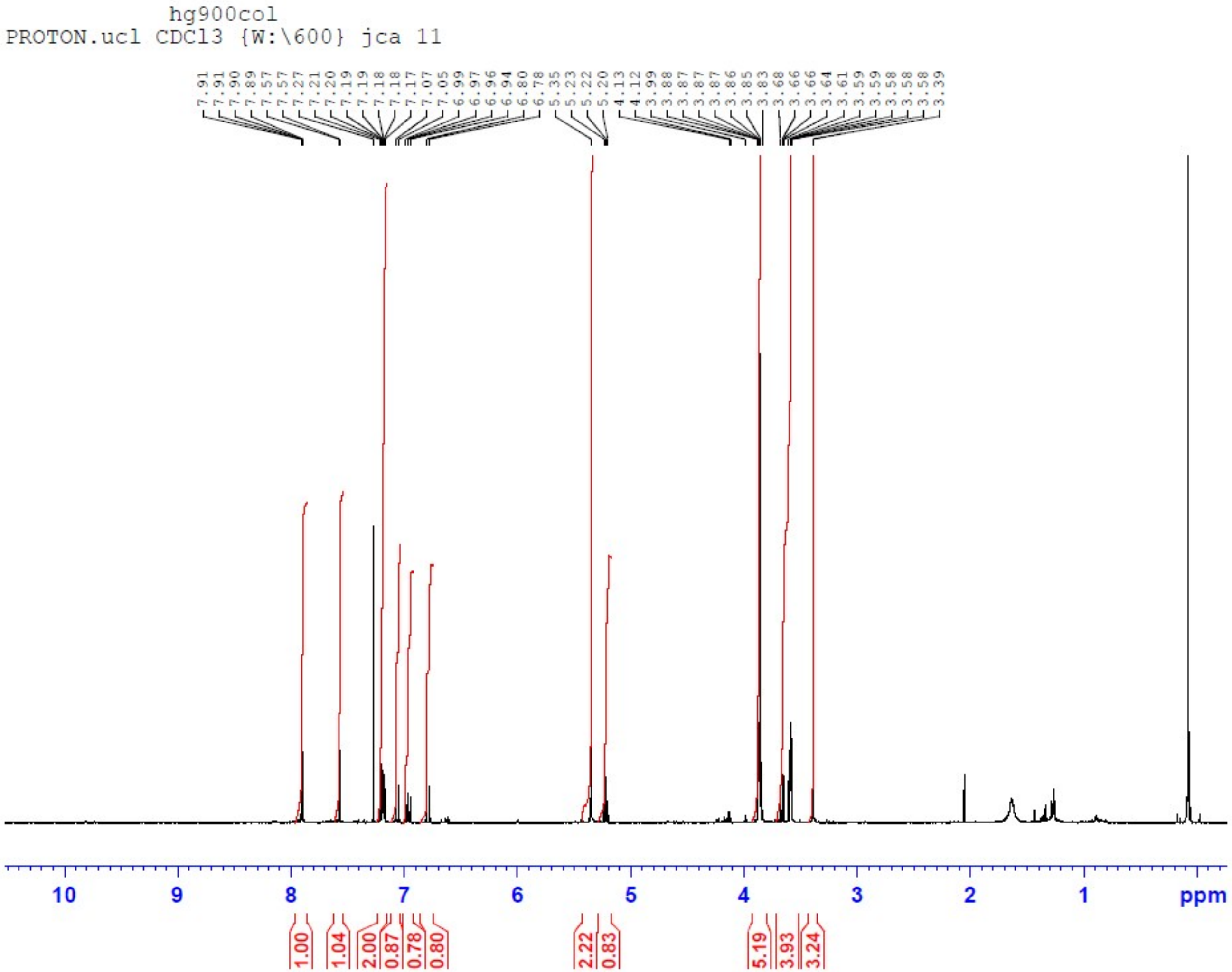

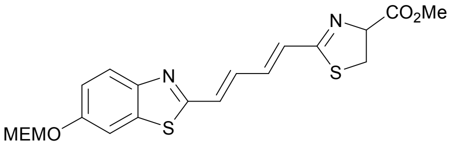

15

```
Current Data Parameters
NAME              HG900col
EXPNO                   10
PROCNO                   1

F2 - Acquisition Parameters
Date_             20160128
Time                 11.28
INSTRUM              AV600
PROBHD   5 mm CPDCH 13C
PULPROG               zg30
TD                   98682
SOLVENT              CDCl3
NS                       4
DS                       0
SWH           12335.526 Hz
FIDRES         0.125003 Hz
AQ            3.9999104 sec
RG                    40.3
DW              40.533 usec
DE               11.48 usec
TE               298.0 K
D1          1.00000000 sec
TD0                      1

======== CHANNEL f1 ========
SFO1        600.1337061 MHz
NUC1                    1H
P1               11.40 usec
PLW1        13.76731014 W

F2 - Processing parameters
SI                  131072
SF          600.1300096 MHz
WDW                     no
SSB      0
LB       0 Hz
GB       0
PC                    1.40
```

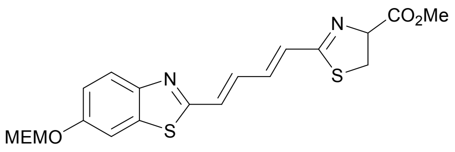
N
CO2Me
S
N
MEMO
S

15

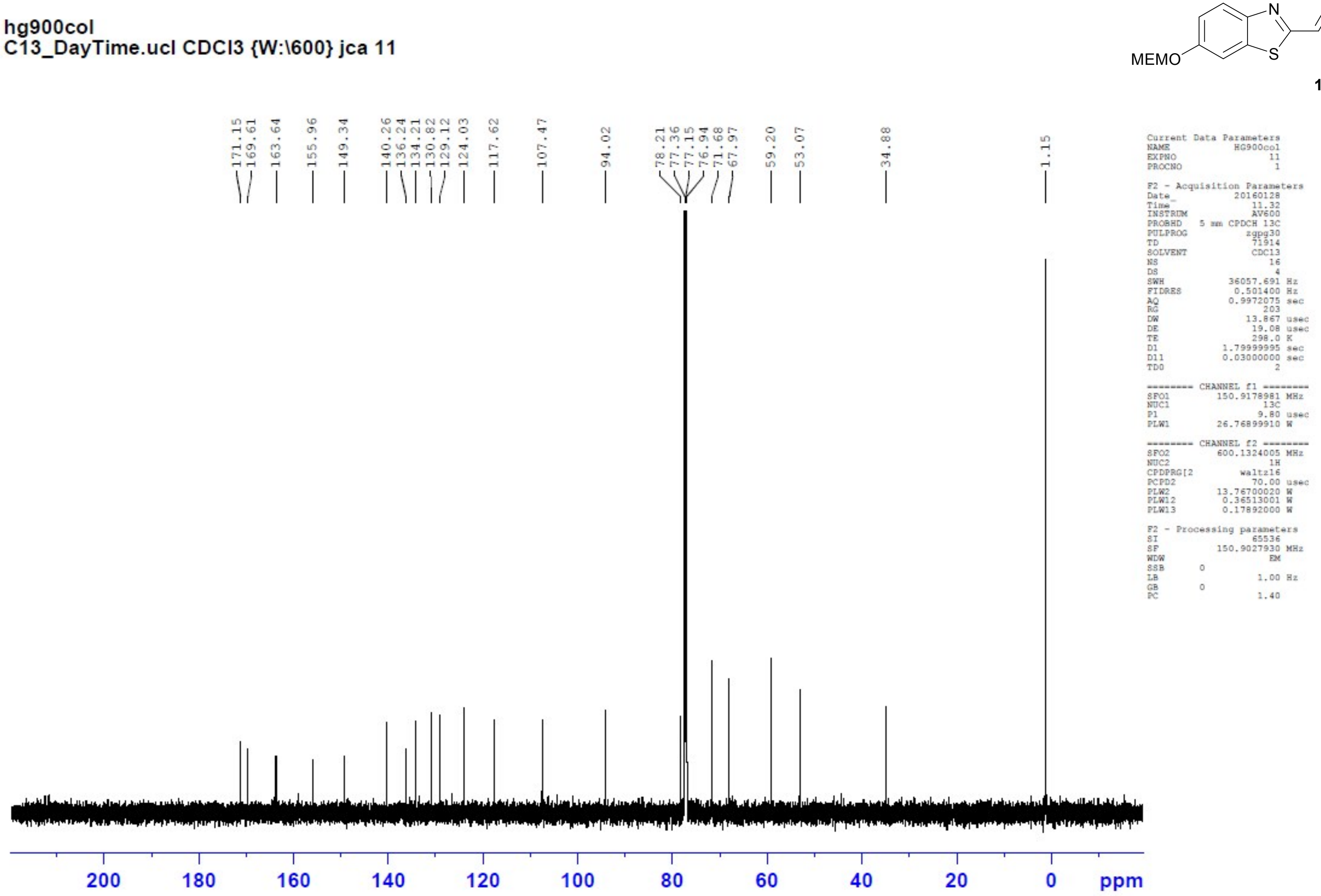
hg900col
C13_DayTime.ucl CDCl3 {W:\600} jca 11
171.15
169.61
163.64
155.96
149.34
140.26
136.24
134.21
130.82
129.12
124.03
117.62
107.47
94.02
78.21
77.36
77.15
76.94
71.68
67.97
59.20
53.07
34.88
1.15
Current Data Parameters
NAME HG900col
EXPNO 11
PROCNO 1
F2 - Acquisition Parameters
Date_ 20160128
Time 11.32
INSTRUM AV600
PROBHD 5 mm CPDCH 13C
PULPROG zgpg30
TD 71914
SOLVENT CDCl3
NS 16
DS 4
SWH 36057.691 Hz
FIDRES 0.501400 Hz
AQ 0.9972075 sec
RG 203
DW 13.867 usec
DE 19.08 usec
TE 298.0 K
D1 1.79999995 sec
D11 0.03000000 sec
TD0 2
======== CHANNEL f1 ========
SFO1 150.9178981 MHz
NUC1 13C
P1 9.80 usec
PLW1 26.76899910 W
======== CHANNEL f2 ========
SFO2 600.1324005 MHz
NUC2 1H
CPDPRG[2 waltz16
PCPD2 70.00 usec
PLW2 13.76700020 W
PLW12 0.36513001 W
PLW13 0.17892000 W
F2 - Processing parameters
SI 65536
SF 150.9027930 MHz
WDW EM
SSB 0
LB 1.00 Hz
GB 0
PC 1.40
200
180
160
140
120
100
80
60
40
20
0
ppm

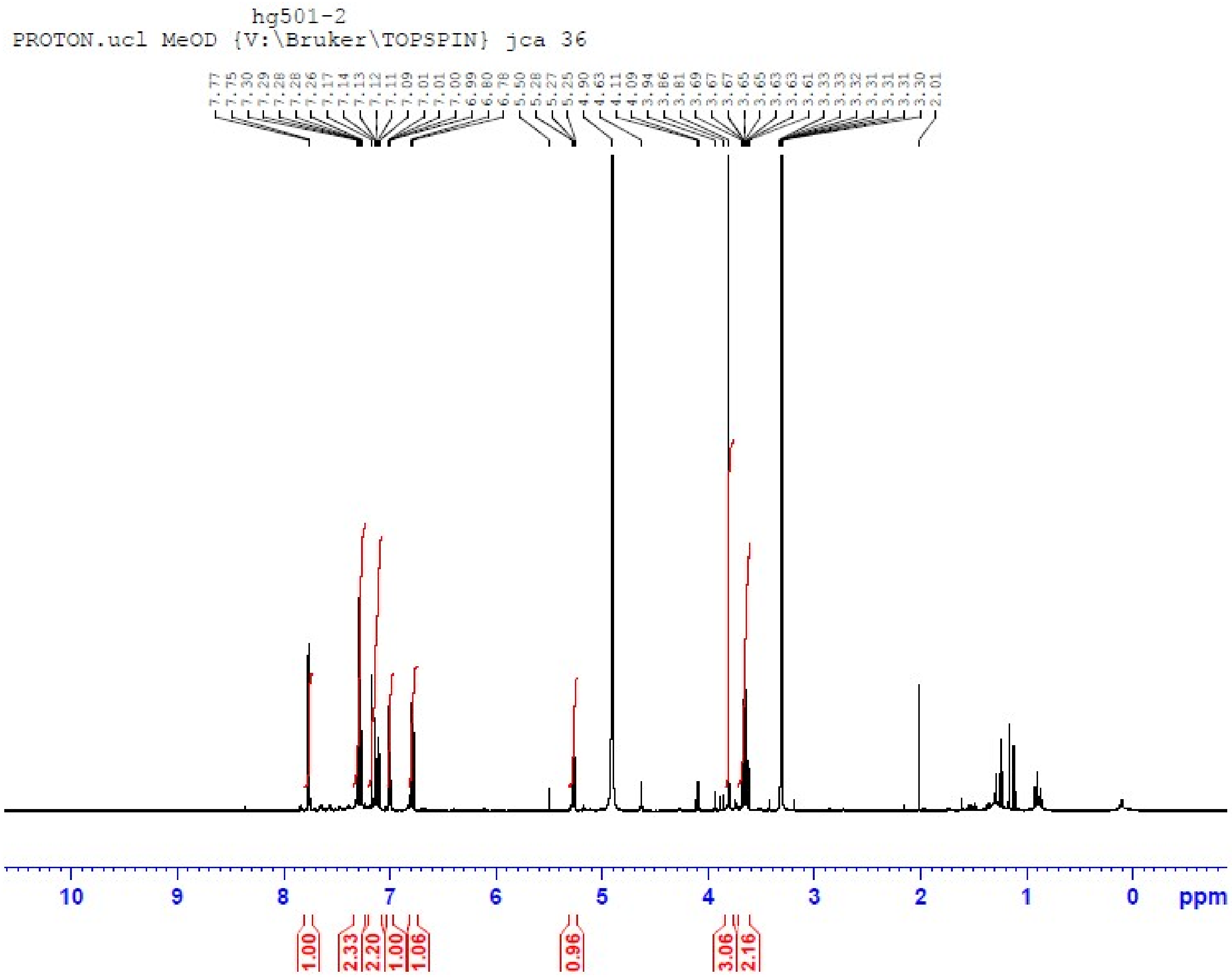

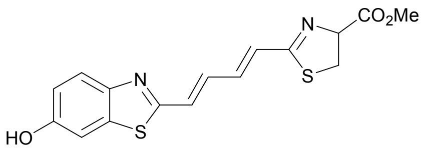

16

```
Current Data Parameters
NAME                NMR
EXPNO                60
PROCNO                1

F2 - Acquisition Parameters
Date_          20120713
Time              11.50
INSTRUM           AV600
PROBHD   5 mm CPDCH 13C
PULPROG            zg30
TD                98682
SOLVENT            MeOD
NS                    8
DS                    0
SWH          12335.526 Hz
FIDRES        0.125003 Hz
AQ           3.9999104 sec
RG                 40.3
DW               40.533 usec
DE                10.48 usec
TE                298.0 K
D1          1.00000000 sec
TD0                   1

======== CHANNEL f1 ========
NUC1                 1H
P1                11.40 usec
PL1                1.00 dB
PL1W        13.76731014 W
SFO1        600.1337061 MHz

F2 - Processing parameters
SI                32768
SF          600.1300141 MHz
WDW                  EM
SSB       0
LB                 0.30 Hz
GB        0
PC                 1.40
```

hg501-2
C13CPD.ucl MeOD {V:\Bruker\TOPSPIN} jca 36

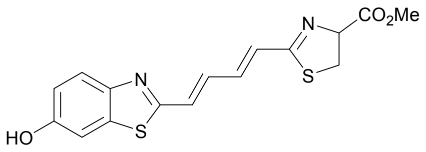

16

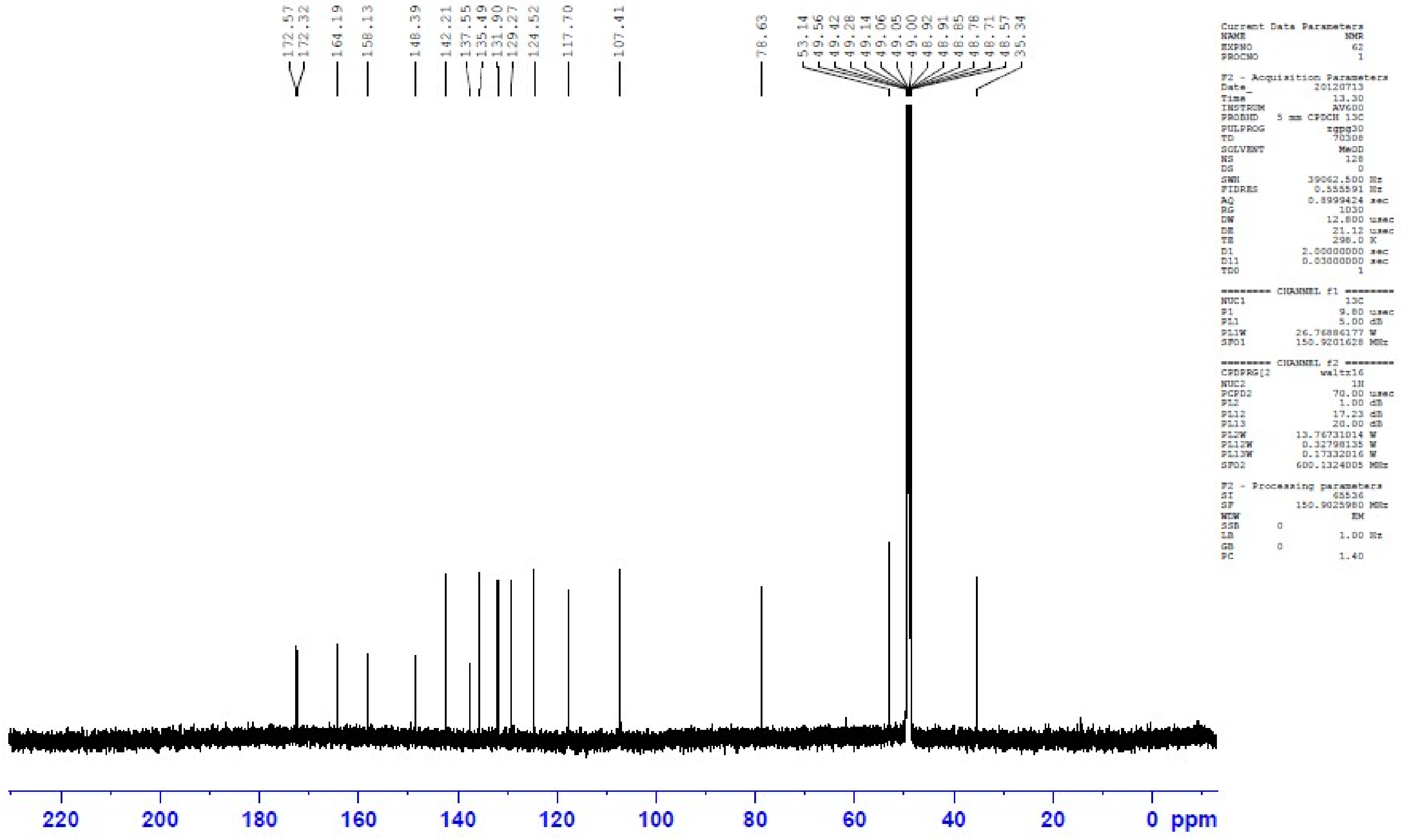

Solvent suppression of largest peak (H_2O).

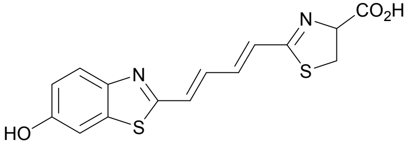

5

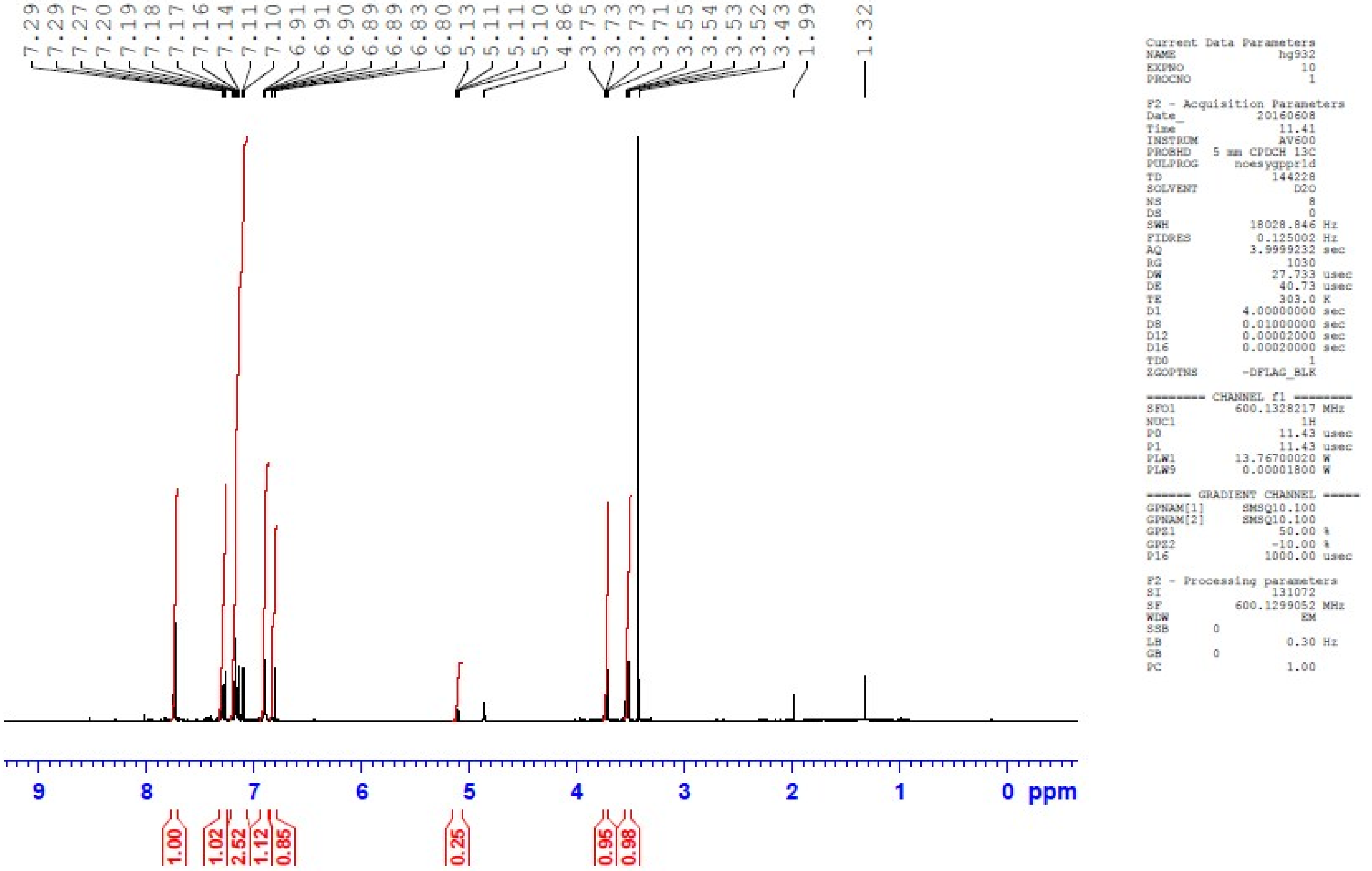

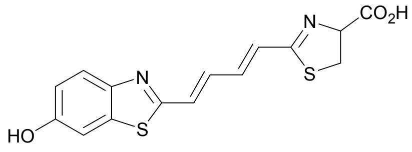

5

C13_DayTime.ucl D2O {W:\600} jca 44

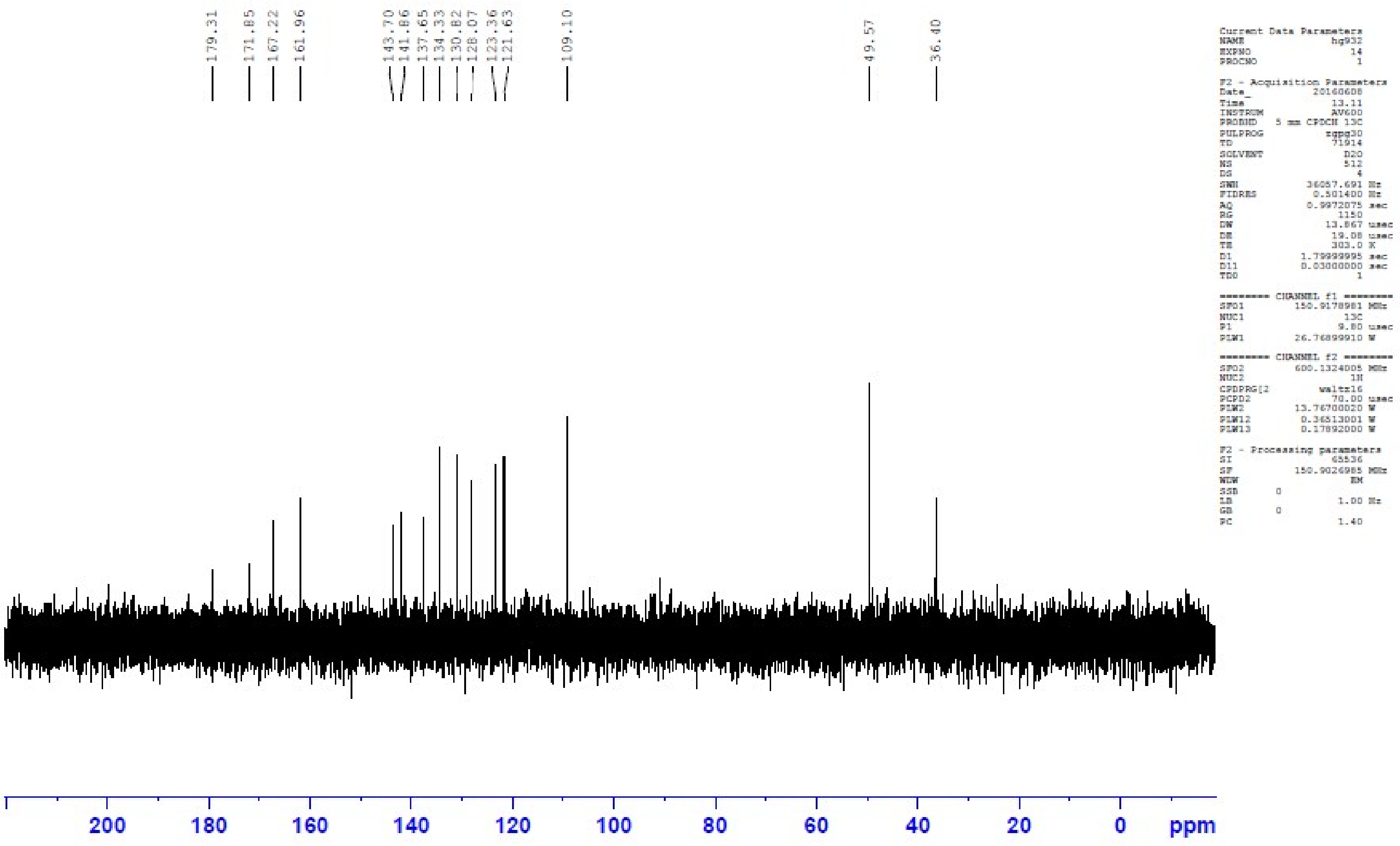

Supplement: Supplementary file 1 [file RA-007-C6RA19541E-s001.pdf]
